# Fungal community succession and major components change during manufacturing process of Fu brick tea

**DOI:** 10.1038/s41598-017-07098-8

**Published:** 2017-07-31

**Authors:** Qin Li, Jianan Huang, Yongdi Li, Yiyang Zhang, Yu Luo, Yuan Chen, Haiyan Lin, Kunbo Wang, Zhonghua Liu

**Affiliations:** 1 Key Laboratory of Tea Science of Ministry of Education, Hunan Agricultural University, Changsha, Hunan 410128 P.R. China; 2 Hunan Provincial Key Laboratory of Crop Germplasm Innovation and Utilization, Hunan Agricultural University, Changsha, Hunan 410128 P.R. China; 3 National Research Center of Engineering Technology for Utilization of Functional Ingredients from Botanicals, Hunan Agricultural University, Changsha, Hunan 410128 P.R. China; 4 Collaborative Innovation Centre of Utilization of Functional Ingredients from Botanicals, Hunan Agricultural University, Changsha, Hunan 410128 P.R. China; 5 Institute of Soil and Water Resources and Environmental Sciences, Zhejiang University, Hangzhou, Zhejiang, 3100058 P.R. China; 6 College of Plant Protection, Hunan Agricultural University, Changsha, Hunan 410128 P.R. China

## Abstract

Fu brick tea is a unique post-fermented tea product which is fermented with microorganism during the manufacturing process. Metabolic analysis showed that most metabolites content were decreased during the manufacturing process of Fu brick tea, except GA (gallic acid). Illumina MiSeq sequencing of ITS gene amplicons was applied to analyze the fungal community succession. The genera *Aspergillus*, *Cyberlindnera* and *Candida* were predominant at the early stage of manufacturing process (from “primary dark tea” to “fermentation for 3 days”), but after the stage of “fermentation for 3 days” only *Aspergillus* was still dominated, and maintain a relatively constant until to the end of manufacturing process. The effects of metabolites on the structure of the fungal community were analyzed by redundancy analysis (RDA) and variation partitioning analysis (VPA). The results indicated that GCG (gallocatechin gallate), EGCG (epigallocatechin gallate) and GA as well as the interactions among them were the most probably ones to influence, or be influenced by the fungal communities during the fermentation process of Fu brick tea. This study revealed fungal succession, metabolite changes and their relationships, provided new insights into the mechanisms for manufacturing process of Fu brick tea.

## Introduction

In general, Chinese teas are divided into six categories according to the processing technology and oxidation degree: green tea (no oxidization), white tea (slightly oxidized), yellow tea (lightly oxidized), oolong tea (partially oxidized), black tea (fully oxidized), dark tea (post-fermented)^1^. These six types of tea have different flavor quality and health benefits because of their characteristic chemical components. The manufacturing process of Fu brick tea involves steaming, piling, pressing, fermentation (microbial growth) and drying. A complex biochemical changes take place during the manufacturing process producing its special flavor and health benefits of Fu brick tea, such as anti-hyperlipidemia, anti-obesity, anti-hyperglycemia, and anti-dysentery activities^2–5^.

Since the quality of Fu brick tea is closely related to the microbial activities during the manufacturing process, microbial community have been intensive studied in the past few years. Microbial counting and identification revealed that genera *Aspergillus*, *Penicillium*, and *Eurotium* were the dominating fungus during the manufacturing process of Fu brick tea^6, 7^. However, traditional microbiological methods, such as cell cultures and colony counting, are of limited value for exploring the variation and structure of microbial communities. Moreover, isolation media may be suitable for only some types of microorganisms, as strains of microorganisms cannot be accurately discriminated based on appearance^8^. Several molecular biology techniques, such as terminal restriction fragment length polymorphism (T-RFLP), polymerase chain reaction denaturing gradient gel electrophoresis (PCR-DGGE), and gene library construction, were used for determining microbial communities and structures^9^. A recent studies using PCR-DGGE analysis revealed that the microorganisms found in Fu brick tea were from or closely related to the genera *Aspergillus*, *Beauveria*, *Debaryomyces*, *Eurotium*, *Pestalotiopsis*, *Pichia*, *Rhizomucor*, and *Verticillium* ^10^, while other studies found that *Aspergillus niger*, *Blastobotrys adeninivorans* and *Bacillus*, *Enterobacteriaceae* were the major fungal and bacterial communities involved in Pu-erh tea, another kind of post-fermentation tea^11, 12^. However, these techniques provide limited information on community information, because only a few sequences can be separated and analyzed. Recently, a high-throughput sequencing technique, also called next generation sequencing technology, was used to investigate microbial composition and diversity in various fermented foods, such as doubanjiang-meju, wine grape and Pu-erh tea^13–15^. Compared to previous culture-dependent or low resolution molecular method (e.g. PCR-DEGG), this technology produces a very large number of reads in a run of many different samples and provides a powerful tool for profiling microbial structure and diversity at genus or even species levels^16, 17^.

The aim of this study was to investigate the fungal community succession, metabolite changes, and assess relationships between fungal community and metabolites during the manufacturing process of Fu brick tea. This knowledge could give new insight into the manufacturing process and provide valuable knowledge to improve the quality of Fu brick tea.

## Results

### Metabolite changes during the manufacturing process of Fu brick tea

**Figure 1:**
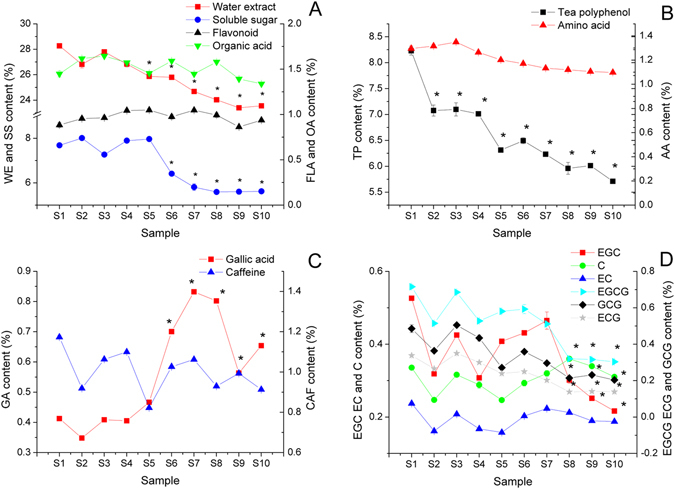
Metabolite changes during manufacturing process of Fu brick tea. water extract (WE), soluble sugar (SS), flavonoid (FLA), organic acid (OA), tea polyphenol (TP), amino acid (AA), gallic acid (GA), caffeine (CAF), epigallocatechin gallate (EGCG), epigallocatechin (EGC), epicatechin gallate (ECG), gallocatechin gallate (GCG), epicatechin (EC), catechins (C). *Indicated that the significance <0.05, compared with S1; **indicated that the significance <0.01, compared with S1.

The metabolites, including water extract (WE), soluble sugar (SS), flavonoid (FLA), organic acid (OA), tea polyphenol (TP), amino acid (AA), gallic acid (GA), caffeine (CAF), epigallocatechin gallate (EGCG), epicatechin gallate (ECG), gallocatechin gallate (GCG), epigallocatechin (EGC), epicatechin (EC), catechins (C), were analyzed during the manufacturing process of Fu brick tea. As shown in Fig. 1, significant decreasing of WE and SS were beginning at S5 and S6, then continued decreased to the end of manufacturing process of Fu brick tea (P < 0.05). A 16.68% decrease in WE and a 26.87% decrease in SS were observed at S10 compared with S1. The level of FLA and OA ranged from 0.86 ± 0.01% to 1.05 ± 0.03% and 1.34 ± 0.20% to 1.65 ± 0.02%, but no significant difference were observed during the manufacturing process of Fu brick tea (Fig. 1A). The concentration of TP was significantly decreased by 30.61% from S1 to S10 during the manufacturing process of Fu brick tea (P < 0.05). But AA ranged from 1.10 ± 0.01% to 1.35 ± 0.02%, which slightly decreased but no significantly difference were observed during the process of Fu brick tea (P > 0.05) (Fig. 1B). The level of CAF ranged from 0.91 ± 0.01% to 1.17 ± 0.01%, which was slightly decreased beginning at S2 and continued to the end of the process (P > 0.05). The level of GA was relatively stable from S1 to S5 and sharply increased from S5 to S7, then decreased until the end of the process, but the concentration also increased by 58.48% in S10, compared with S1 (P < 0.05) (Fig. 1C). The level of EGC was slightly decreased from S1 to S8 and dramatically decreased from S9 to S10 and a 58.83% decrease was observed at S10 compared with S1 (P < 0.05). But the concentration of C and EC ranged from 0.25 ± 0.01% to 0.34 ± 0.01% and 0.16 ± 0.01% to 0.24 ± 0.01%, which were relatively stable during the manufacturing process. The level of EGCG, GCG and ECG were slightly decreased from S1 to S7 (P > 0.05), and significantly decreased from S8 to the end of the manufacturing process of Fu brick tea (P < 0.05) (Fig. 1D).

### Diversity of fungal communities during the manufacturing process of Fu brick tea

**Figure 2:**
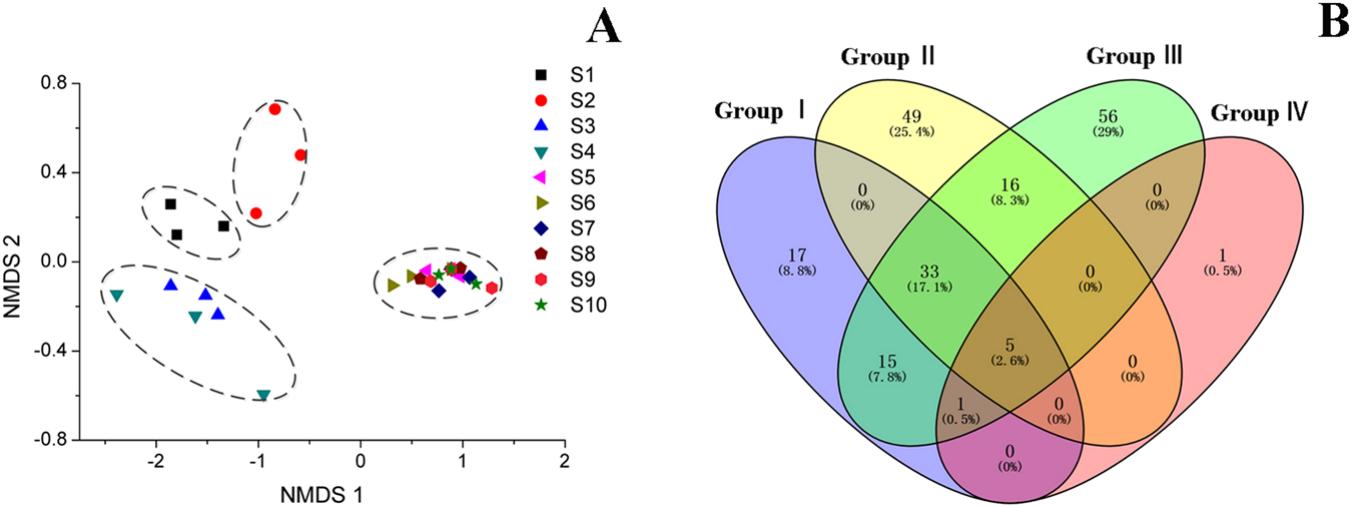
NMDS (**A**) and Venn diagram analysis (**B**) of fungal communities in samples during manufacturing process of Fu brick tea.

After quality filtering and chimera removal, a total of 285,184 of valid sequences were generated from the samples, with an average sequence number of 28,518 for each sample (range from 20846 to 37278). At 97% sequence identity, 216 OTUs (operational taxonomic units) were identified (Supplementary Information Table S1), ranged from 3 to 130 through the manufacturing process. The value of Good’s coverage were >99% for all sequences from ten groups, indicating sufficient sequencing depth for microbial communities in Fu brick tea. The Shannon and Simpson index, the Chao1 and ACE estimator demonstrated that both the diversity and richness of fungus were no significant change from S1 to S4 sample, but dramatically decreased in S5 and maintained relatively constant until the end of the manufacturing process of Fu brick tea (Supplementary Information Table S2). These changes were related to the dominance of specific taxonomic group during the manufacturing process. The rarefaction curves and Shannon-Wiener curves for all samples almost reached the saturation phase, suggesting that there few new microbes would be identified by increasing the sequence depth, and the majority of fungal microbes in the samples had already been captured in the analysis (Supplementary Figure S1A and B). Rank-abundance curves indicated that a majority of the reads belonged to rare organisms represented by only a few sequences, particularly for S5~S10 samples (Supplementary Figure S2). To evaluate the similarities of fungal communities in different process stage of Fu brick tea samples, NMDS analysis based on Bray-Curtis distance was performed. The result showed that the samples could be divided into four groups: the first group, including S1; the second group, including S2; the third group, including S3, S4; the fourth group, including S5~S10 (Fig. 2A). The Venn diagram analysis was used to show the shared OTUs among different stages of manufacturing process after singleton sequences being removed. The samples were divided into four groups based on the NMDS analysis. This analysis showed that only 5 of the identified OTUs were shared among all the groups, while 38 of 174 OTUs, 54 of 229 OTUs and 6 of 133 OTUs were shared between the groups in group I and group II, group II and group III, group III and group IV, respectively (Fig. 2B). The detail information of Venn diagram analysis was provided in Supplementary Information Table S3.

### Composition of fungal communities during the process of Fu brick tea

**Figure 3:**
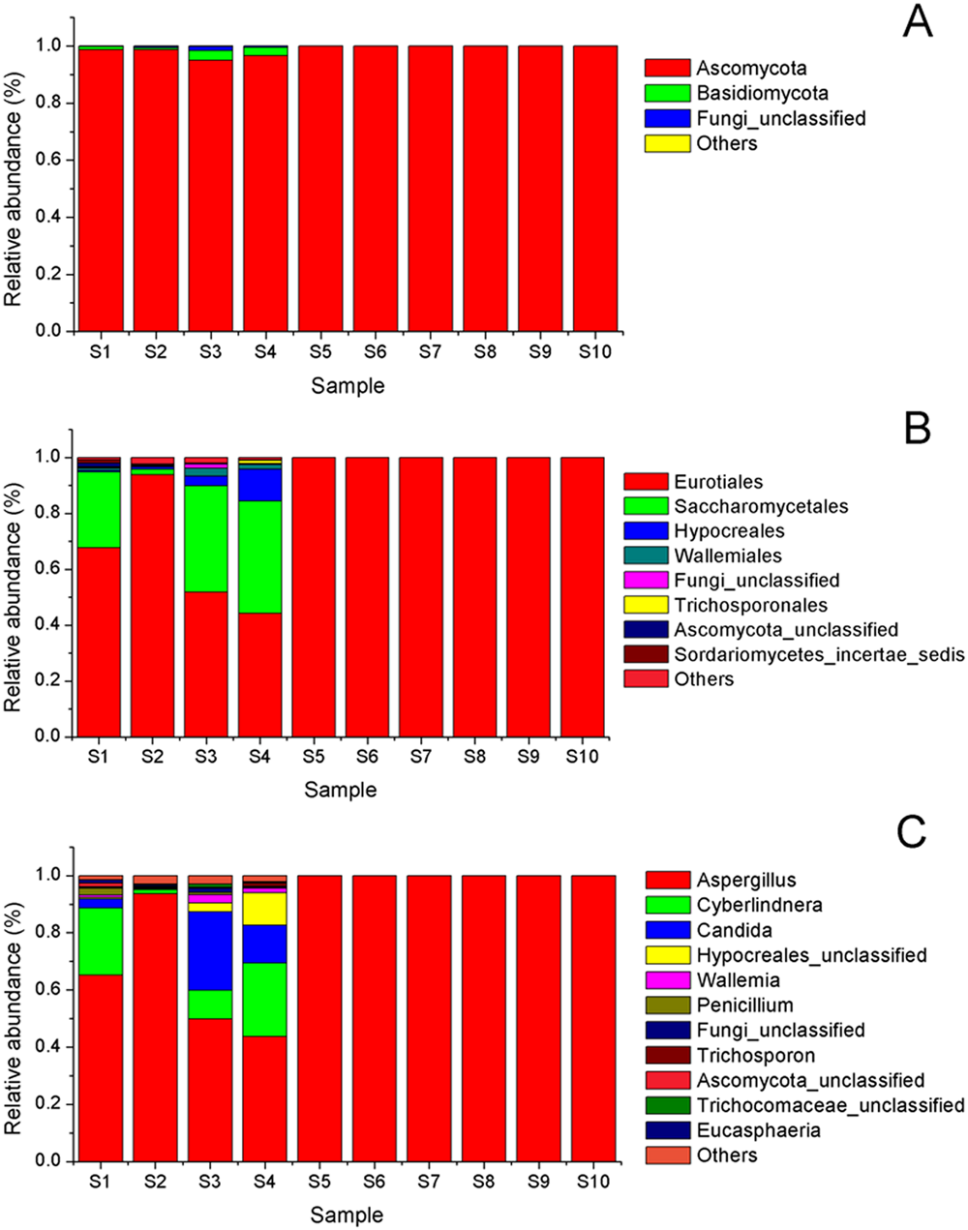
Fungal taxonomic compositions showing the fungal successions at phylum (**A**), order (**B**) and genus (**C**) level during manufacturing process of Fu brick tea. The taxonomic abundance <1% were classified into “others”. Phylum level (**A**), order level (**B**), genus level (**C**).

To identify the taxonomic composition of fungal communities among three groups, the RDP classifier was used to assign the sequence tags to different taxonomic levels at 70% threshold. All the identified OTUs could be assigned into 4 different phyla, 16 classes, 40 orders, 69 families or 102 genera. As shown in Fig. 3, Ascomycota was the predominant phylum in all samples, accounting for 95.05%~99.99% of total effective sequences. *Basidiomycota* and some other phylum fungi were also identified in the early stage of process samples, but almost not detected after S5 of manufacturing process (Fig. 3A). The order *Eurotiales*, *Saccharomycetales* and *Hypocreales* predominant from S1 to S4 samples, but quickly replaced by *Eurotiales* after S5 and remained stable until the end of manufacturing process in Fu brick tea (Fig. 3B).

At the genus level, *Aspergillus*, *Cyberlindnera* and *Candida* comprised >80% of all sequence. *Aspergillus* dominated the whole manufacturing process of Fu brick tea, which represented the largest fraction, first increased from 65.13% in S1 sample to 93.58% in S2 sample and significantly decreased to 43.63% in S3, then dominant until the end of process, with relative abundance clearly increased to 99.99% in S6. *Cyberlindnera* account for 23.46% of all sequences in S1 and was dramatically decreased to 1.47% in S2, then sharply increased to 25.72% in S3, and almost not detected from S6 sample to the end of manufacturing process. *Candida* increased from the initial 3.25% in S1 to 27.46% in S2 and decreased to 13.23% in S3, but also not detected from S6 to the end of manufacturing process. Variations in some small proportions in the groups *Wallemia* (0.81% to 0.57%), *Penicillium* (2.49% to 0.01%) were also observed during manufacturing process of Fu brick tea (Fig. 3C).

### Relationships between fungal community and metabolites

**Figure 4:**
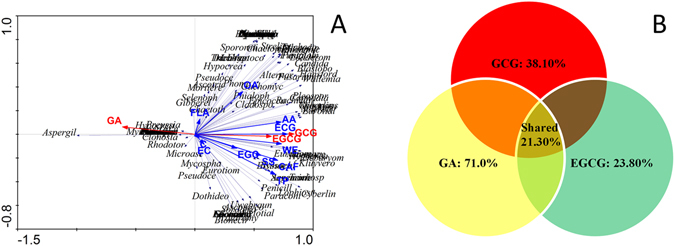
RDA and VPA showing the correlations between fungal structure and metabolites concentrations during manufacturing process of Fu brick tea. Ordination diagram of RDA (**A**). Variance decomposition of VPA (**B**). Gallic acid (GA), epigallocatechin gallate (EGCG), gallocatechin gallate (GCG). Red arrow indicated the metabolite variance significant effect on the fungal community structure (P < 0.05).

In order to evaluate the effects of metabolite factors on the structure of fungal community during manufacturing process of Fu brick tea, the multivariate analysis were performed. The gradient lengths were 0.959, 0.652, 0.186 and 0.183 from the first to the fourth axis, as retrieved by detrended correspondence analysis (DCA). The result of DCA indicated that the gradient length of the first axis was less than 3SD. Therefore, the linear model with RDA was considered to be the appropriate ordination method for direct gradient analysis. As shown in Fig. 4, the results of forward selection of RDA showed that the most variation of the fungal community structure could be well explained by GCG, EGCG and GA during the manufacturing process. The model statistically explained up to 90.30% of the variation (P = 0.004) (Fig. 4A). Furthermore, the result of VPA indicated that GCG, EGCG and GA explained 38.10% (P = 0.002), 23.80% (P = 0.004) and 7.10% (P = 0.026) of the variation in fugal community structure, respectively. The variation shared by GCG, EGCG and GA catechins was 21.30% (Fig. 4B). Those factors as well as the interactions among them were the most probably ones to influence, or be influenced by the fungal communities during the fermentation process of Fu brick tea.

As shown in Table 1, Pearson’s correlation between fungal community and major metabolites were also analyzed. Genus of *Aspergillus* was significantly (P < 0.05) correlated with WE, TP, AA, CAF, EGCG, GCG and ECG. *Cyberlindnera* was significantly (P < 0.05) correlated with WE, TP, CAF, GCG and ECG. *Candida* was significantly (P < 0.05) correlated with AA, GCG and ECG. *Wallemia* and *Fungi_unclassified* were significantly (P < 0.05) correlated with WE, AA, GCG and ECG. *Penicillium* was significantly (P < 0.05) correlated with WE, TP, CAF, EGC, EGCG, GCG and ECG. *Ascomycota_unclassified* was significantly (P < 0.05) correlated with TP and CAF. *Eucasphaeria* was significantly (P < 0.05) correlated with TP and CAF. However, no fungal genera were significantly (P > 0.05) correlated with FLA, OA, EC and C.

**Table 1:** Pearson’s correlation between the relative abundances of important genera (relative abundance >1%) and metabolite variables^a^.

	WE	FLA	TP	AA	SS	CAF	GA	OA	EGC	DL-C	EC	EGCG	GCG	ECG
*Aspergillus*	−0.735*	−0.044	−0.682*	−0.737*	−0.564	−0.697*	0.618	−0.415	−0.352	−0.028	−0.072	−0.649*	−0.840**	−0.785**
*Cyberlindnera*	0.696*	−0.045	0.774**	0.601	0.565	0.754*	−0.563	0.180	0.398	0.079	0.154	0.603	0.755*	0.704*
*Candida*	0.569	0.09	0.390	0.657*	0.355	0.490	−0.463	0.516	0.253	0.045	0.059	0.543	0.706*	0.657*
*Hypocreales_unclassified*	0.368	0.359	0.284	0.387	0.427	0.296	−0.408	0.337	−0.031	−0.140	−0.300	0.240	0.473	0.434
*Wallemia*	0.674*	0.04	0.531	0.749*	0.456	0.578	−0.560	0.524	0.318	0.024	0.074	0.625	0.795**	0.744*
*Penicillium*	0.704*	−0.446	0.874**	0.575	0.424	0.825**	−0.464	0.001	0.641*	0.243	0.534	0.697*	0.692*	0.655*
*Fungi_unclassified*	0.636*	−0.032	0.463	0.780**	0.426	0.427	−0.558	0.597	0.288	−0.071	0.029	0.589	0.721*	0.687*
*Trichosporon*	0.44	0.285	0.411	0.436	0.505	0.360	−0.474	0.301	0.030	−0.158	−0.273	0.292	0.515	0.478
*Ascomycota_unclassified*	0.607	−0.462	0.824**	0.447	0.370	0.758*	−0.381	−0.115	0.604	0.246	0.532	0.602	0.568	0.539
*Trichocomaceae_unclassified*	0.491	0.078	0.288	0.601	0.269	0.402	−0.391	0.514	0.221	0.051	0.067	0.487	0.625	0.582
*Eucasphaeria*	0.519	−0.469	0.757*	0.337	0.295	0.711*	−0.293	−0.202	0.578	0.277	0.556	0.530	0.472	0.447

^a^Water extract (WE), soluble sugar (SS), flavonoid (FLA), organic acid (OA), tea polyphenol (TP), amino acid (AA), gallic acid (GA), caffeine (CAF), epigallocatechin gallate (EGCG), epigallocatechin (EGC), epicatechin gallate (ECG), gallocatechin gallate (GCG), epicatechin (EC), catechins (C). *Indicated that the significance <0.05; **indicated that the significance <0.01.

## Discussion

Fu brick tea is a unique post-fermented tea product which is fermented with microorganism during the manufacturing process. To the best of our knowledge, this is the first report on the fungal community succession and metabolite changes during the manufacturing process of Fu brick tea by high throughput Illumina MiSeq sequencing, spectrophotometry and HPLC. The results, from dramatic changes in fungal community structure and metabolites as well as correlation analysis, indicated strong relationships between metabolites and fungal community during the manufacturing process of Fu brick tea.

Our metabolic analysis showed that WE, SS, TP and catehins were significantly decreased, but GA was significantly increased during the manufacturing process of Fu brick tea. These changes probably resulted from moisture and heat reaction and microbial metabolism^18^. Several enzymes secreted from genus of *Aspergillus*, such as cellulases, hemicellulases, proteases and α-amylases, could catalyze the major metabolites in Pu-erh tea during fermentation process^11, 19^. The decrease of SS may be related to microbial growth that SS could be used as the carbon source of microbial growth during the manufacturing process^20^. Our previous study indicated that the degradation of TP and catechins in the early stage of process might be resulted from moisture and heat reaction, but in the later stage of process was mainly related to the microbial metabolism. And this degradation was good for the formation of Fu brick tea’s taste and its special aroma^20^. The aldehyde compounds of stale aroma and terpene alcohols of flower aroma which specially increased after the fermentation process were from decarboxylation and oxidative deamination of amino acids^21^. The increase of GA may be also the result of hydrolysis of galloylated catechins and microbial metabolism, which might be helpful on some bioactivity of Fu brick tea^10^. Interesting, these decreased components were the major functional components of tea, but Fu brick tea still has a strong health benefits. Mo *et al*. reported that the activities increased with the course of microbial fermentation process (MFP) of Fu brick tea, which indicated that this process produced new varieties of functional metabolites^2^. Furthermore, new metabolites such as three triterpenoids^22^, one norisoprenoid^23^, seven new B ring fission catechins (flavan-3-ols) derivatives^24–26^ were reported from a few phytochemical investigations on Fu brick tea. Especially these B ring fission catechins have not been identified in other tea products, which suggested that the biochemical profiles of Fu brick tea were influenced significantly by MFP.

Fungal community succession showed that the genera *Aspergillus*, *Cyberlindnera* and *Candida* were predominant from S0 to S4, but only the genus of *Aspergillus* was still dominated in Fu brick tea from S5 and maintained constant until the end of manufacturing process. The genus *Aspergillus* is a group of filamentous fungi consist of more than 250 species, which is the most economically important of the fungal genera^27^. Many species of *Aspergillus* are used in biotechnology for the production of various metabolites, such as antibiotics, organic acids, medicines or enzymes, or as agents in many food fermentations^28^. The fungal genus *Eurotium*, which is the teleomorph of *Aspergillus*, has been proved to be a rich source of novel bioactive metabolites^29, 30^. Since the recent synonymization of the teleomorph-based genus *Eurotium* with *Aspergillus* by the International Commission on *Aspergillus* (ICPA, 2012), which adopted the newly established principle “one fungus, one name” (Norvell, 2011), species formerly included in the genus *Eurotium* are displayed with their *Aspergillus* name^27^. Many previous reports showed that *Aspergillus cristatum* (*Eurotium cristatum*) was the dominant fungus during the manufacturing process of Fu brick tea^31, 32^, and it is considered to be safe under low- and high-osmolarity conditions by genomic, transcriptomic and HPLC analysis^33^. Several indole alkaloids and indole diketopiperazine alkaloid were identified from the culture extract of *Aspergillus cristatum* and these compounds were proved to possess brine shrimp lethality, antibacterial activity against E. *coli*, radical scavenging activity against DPPH radicals, and exhibited marginal attenuation of 3T3L1 pre-adipocytes^34–36^. Both emodin and physcion were also identified in Fu brick tea fermented by *Aspergillus cristatum* ^10^. Non-mycotoxigenic strains of *Aspergillus repens* and *Aspergillus rubrum* are used as starter cultures in the manufacture of the traditional fermented food *katsuobushi*, from bonito (*Katsuwonuspelamis*), in Japan^37^. During *katsuobushi* fermentation these xerophilic fungi produce effective antioxidants, which participate in the suppression of lipid oxidation such that the fermented *katsuobushi* is resistant to lipid oxidation and has a long shelf-life^38^.

The genus *Lindnera* was established to accommodate some *Pichia* species in the *Pichia anomala* clade^39–41^. But *Lindnera* is a later homonym of a valid published plant genus, the genus *Cyberlindnera* was introduced as a replacement name for *Lindnera*, with 21 new combinations^42^. The *Cyberlindnera* clade contains 23 recognized teleomorphic species and 12 *Candida* species are also related to this clade^43^. The yeast *Cyberlindner ajadinii*, previously referred to as *Pichia jadinii*, *Hansenul ajadinii* or *Candida guilliermondii*, was reported to be the teleomorph of *Candida utilis* (also referred to as *Torula* yeast)^44^, which was used for almost one hundred years for various biotechnological applications^45^. Because of its capability to efficiently assimilate pentose, including xylose and arabinose, and its robust fermentation characteristics, *Cyberlindner ajadinii* was exploited to grow large amounts on waste hardwood hydrolysates from the pulp industry. Moreover, some species of *Cyberlindnera* and *Candida*, which had a high capability of tannin tolerance, were also isolated from Miang (a fermented food product prepared from tea leaves)^46^. The genus *Candida* has become one of the largest in species number, present in almost every environment. Yeasts of this genus are abundantly distributed in nature on land and sea, associated with animals or plants and inanimate objects^47^. This genus is distributed across the *ascomycetous* yeast domain, overlapping with other genera, according to phylogenetic analysis using ribosomal genes. Some species of *Candida* genus were used in food fermentation, such as *Candida etchellsii*, *Candida milleri, Candida rugosa* and *Candida tropicalis* ^28^. Some species of *Candida* has high phenol degradation ability^48–51^, bioconversion of corn fiber, sugarcane bagasse and xylose into the sweetener xylitol^52, 53^, enhance of clove and smoked favors and biotransformation of maltose into ethanol in soy sauce process^54^. These properties of yeast *Cyberlindnera* and *Candida* genera indicate that their enzyme activities improve the quality of Fu brick tea by degradation of phenol, producing the sweet substance xylitol, and other favors flavour substance. The yeast genera may play an indispensable role in the initial stage of Fu brick tea fermentation.

In conclusion, predominant fungus and major metabolites were analyzed using Illumina MiSeq sequencing, spectrophotometry and HPLC methods, respectively. The metabolic analysis showed that most metabolites content were decreased during the manufacturing process of Fu brick tea, except GA. Illumina MiSeq sequencing analysis showed that the genera *Aspergillus*, *Cyberlindnera* and *Candida* were dominant at the early stage of manufacturing process, but after the stage of “fermentation for 3 days” only the genus Aspergillus was still dominated and maintain a relatively constant until to the end of manufacturing process of Fu brick tea. In addition, the effects of metabolites on the structure of the fungal community were analyzed by RDA and VPA. The results indicated that GCG, EGCG and GA as well as the interactions among them were the most probably ones to influence, or be influenced by the fungal communities during the fermentation process of Fu brick tea.

## Materials and Methods

### Tea leaf samples and process characterization

Sampling of Fu brick-tea was carried out in a major Fu brick tea production factory, Yiyang Fu Cha Industry Development Co. Ltd. (Yiyang, Hunan Province, China). Primary dark tea, used as raw materials for the process of Fu brick tea, was manufactured from fresh tea leaves as described by DB43/T660-2011 (Supplement Material 1)^55^. And the manufacturing process of Fu brick tea was descripted as follow: a 100 kg primary dark tea was moistened by steaming then piled up to 1.5 m in height and subjected to temperatures as high as 80 °C overnight. After pile fermentation, the tea materials were mixed with 15 L of tap water, then partitioned and pressed into desired sizes of brick tea. And the brick teas were placed in the fermentation room for 20~22 days. During the fermentation process, the temperature and relative humidity of fermentation room was 30 ± 2 °C and 75 ± 3% from 0 day to 15 day. Then the temperature of fermentation room was increased by 2 °C per day until the end of the manufacturing process during the drying process. Finally, the tea products were packaged and stored. Three independent batches of Fu brick tea were prepared simultaneously by the same manufacturing process. The samples were collected from three independent batches of Fu brick tea at different stage of manufacturing process: primary dark tea (S1), piling-fermentation tea materials (S2), fermenting brick-tea in the fermentation room for 0 days (S3), 3 days (S4), 6 days (S5), 9 days (S6), 12 days (S7), 15 days (S8), 18 days (S9) and newly made tea products (S10) at day 22. The samples were packaged in sterile polyethylene bags, transported to the laboratory and stored at −80 °C until required.

### Chemical analysis

The content of WE and OA was analyzed determined as described by GB/T 8305-2013 and GB/T 12456-2008, respectively^56, 57^. The concentration of TP, and AA in tea leaves were determined using the spectrophotometric method based on Folin-Phenol and the ninhydrin assay as described by GB/T 8313-2008 and GB/T 8314-2013, respectively^58, 59^. The content of GA, CAF, EGCG, ECG, GCG, EGC, CEC and C were determined, as described previously^60^. The concentration of FLA and SS were determined by a colorimetric method, as described previously^61, 62^. All analysis was performed in three times. The detailed approaches are described in Supplementary Material 2.

### DNA extraction and Illumina MiSeq sequencing

Each tea sample (25 g) was mixed with sterile water (125 mL), stirred thoroughly, filtered through three layers of coarse sterile gauze to remove large particles, and centrifuged at18,000 g for 10 min at 4 °C. The microbial genomic DNA was extracted by the E.Z.N.A.Soil DNA Kit (Omega Bio-tek, Norcross, GA, US), and the procedure was according to the manufacturer’s protocols.

The ITS1 region of fungal was amplified by PCR for MiSeq sequencing. The ITS1 region of fungi was amplified with the forward primer (5′-CTTGGTCATTTAGAGGAAGTAA-3′) and the reverse primer ITS2 (5′-GCTGCGTTCTTCATCGATGC-3′)^63^. Different barcode sequences were added at the 5′ end of the forward primer for multiplexed pyrosequencing. PCR were carried out in a 20 μL reaction volumes containing 10 ng DNA template, 2 μL dNTPs (2.5 mM), 0.4 μL of each primer (5 μΜ) and0.4 UμL FastPfu Polymerase (Applied Biosystems) in the appropriate 56 FastPfu Buffer (4 μL) and de-ionized ultrapure water (to 20 μL). The protocol was optimized with low cycles for better accuracy and reliability of the subsequent data analysis. The PCR condition were initial denaturation at 95 °C for 2 min, followed by 25 cycles of denaturation at 95 °C for 30 s, annealing at 55 °C for 30 sand extension at 72 °C for 45 s, with a final extension phase at 72 °C for 10 min. The PCR products were extracted from 2% agarose gels and purified using the AxyPrep DNA Gel Extraction Kit (Axygen Biosciences, Union City, CA, US) according to the protocol and quantified using QuantiFluor™-ST (Promega, U.S.). Samples were then pooled at equal concentrations. Parallel tagged sequencing was performed using Illumina Miseq Sequencing in Majorbio Bio-Pharm Technology Co., Ltd., Shanghai, China.

### Data processing and statistical analyses

Raw reads were demultiplexed and quality-filtered using Trimmomatic software (version 0.36, http://www.usadellab.org/cms/?page=trimmomatic) with the following criteria^64^: (a) The 250 bp reads were truncated at any site receiving an average quality score less than 20 over a 10 bp sliding window, discarding the truncated reads that were shorter than 50 bp; (b) Exact barcode matching, two nucleotide mismatch in primer matching, reads containing ambiguous characters were removed; (c) Only sequences that overlap longer than 10 bp were assembled according to their overlap region. Reads that could not be assembled were also discarded. These sequences were clustered to OTUs (operational taxonomic units) at 97% sequence identity by using Mothur (version 1.25.0, http://www.mothur.org)^65^. The OTUs were used for alpha diversity (Shannon and Simpson), richness (ACE and Chao1), Good’s coverage, Rarefaction curve, Shannon-Wiener curve and Venn diagram were performed by Mothur^65^. In addition, Nonmetric Multidimensional Scaling (NMDS) diagram and rank_abundance curves were generated by using R package vegan (version 3.4.0, https://mirrors.tuna.tsinghua.edu.cn/CRAN/). CANOCO 4.5 software was used to conduct multivariate analysis using detrended correspondence analysis (DCA), redundancy analysis (RDA) and variation partitioning analysis (VPA)^66^. One-way ANOVA test, Pearson’s correlation coefficients and P values were calculated with IBM SPSS 19.0 (https://www.ibm.com/analytics/us/en/technology/spss/). All results are presented as the mean value (±SE). Differences between groups were declared significant at P < 0.05.

## Electronic supplementary material

Supplementary infromation
