# Supplementary material for: Fungal community succession and major components change during manufacturing process of Fu brick tea
Source: Sci Rep. 2017 Jul 31;7:6947. doi: 10.1038/s41598-017-07098-8 (PMC5537287; doi:10.1038/s41598-017-07098-8)
Supplement: Supplementary file 1 — Supplementary infromation [file 41598_2017_7098_MOESM1_ESM.doc]

**Supplementary Information**

**Fungal community succession and major components change during manufacturing process of Fu brick tea**

Qin Li ^1,2,3,4^, Jianan Huang^1,2,4^, Yongdi Li ^1^, Yiyan Zhang^1^, Yu Luo^5^, Yuan Chen^6^, Haiyan Lin^3^, Kunbo Wang^1,3,4^, Zhonghua Liu^2,3,4^*

1. Key Laboratory of Tea Science of Ministry of Education, Hunan Agricultural University, Changsha, Hunan 410128, P.R. China

2. Hunan Provincial Key Laboratory of Crop Germplasm Innovation and Utilization, Hunan Agricultural University, Changsha, Hunan 410128, P.R. China

3. National Research Center of Engineering Technology for Utilization of Functional Ingredients from Botanicals, Hunan Agricultural University, Changsha, Hunan 410128, P.R. China

4. Collaborative Innovation Centre of Utilization of Functional Ingredients from Botanicals, Hunan Agricultural University, Changsha, Hunan 410128, P.R. China

5. Institute of Soil and Water Resources and Environmental Sciences, Zhejiang University, Hangzhou, Zhejiang 3100058, P.R. China.

6. College of Plant Protection，Hunan Agricultural University, Changsha, Hunan 410128, P.R. China

*ZHL: zhonghua-liu@hotmail.com

Tel: +86-731-84635306

Fax: +86-731-84635304

**Supplementary material 1**

Detail process of primary dark tea

Primary dark tea was processed from fresh tea leaves according to the “The technique specification for raw Anhua dark tea processing (DB43/T660-2011)”. The sequence of manufacturing process was as followed: fresh tea leaves → fixing → rolling → piling → drying (primary dark tea). Mature tea leaves (one bud and three-six leaves) plucked from medium and sprinkled with water then fixing at 280~320℃. The fixing leaves rolled twice by machine. Then the rolling leaves piled 1m high for 12-24h, with leaves temperatures of 50℃ to 60℃ and moisture content of 65% to 68%. In old days, the pile fermentation leaves were dried in Seven Stars Oven with pinewood (70℃). Nowadays auto drying machine as those employed in green tea manufacture were used to do it.

**Supplementary material 2**

Detailed of chemical analysis methods

The content of WE and OA was analyzed determined as described by GB/T 8305-2013 and GB/T 12456-2008, respectively. The concentration of TP, and AA in tea leaves were determined using the spectrophotometric method based on Folin-Phenol and the ninhydrin assay as described by GB/T 8313-2008 and GB/T 8314-2013, respectively. The content of GA, CAF, EGCG, ECG, GCG, EGC, CEC and C were determined by HPLC analysis, as described previously[^5^](#_ENREF_5). Separation was completed using the Welchrom C18 column (4.6 mm i.d. × 250 mm). The mobile phases were eluted in solvent A (0.02 mol/L K_2_H_3_PO_4_) and solvent B (100% acetonitrile). Elution conditions and flow rates were: in 0~15 min solvent B was kept at 13%, at 15~25 min solvent B linearly increased from 13% to 20% and kept at 20% for 5 min, then decreased to 13% in 2 min. The flow rate was 1.0 mL/min. The temperature of the column oven was maintained at 30°C. The injection volume was 10 μL. The chemical compounds were identified in the tea liquids by comparing the retention times of the peaks with those of the standards. The concentration of FLA and SS were determined by a colorimetric method, as described previously. All analysis was performed in three times.

**Reference**

1 Zhou, W., Xu, J., Lu, X., Wang, J. & Sha, H. in *Tea-Determination of water extract content* Vol. GB/T 8305-2013 (China Agriculture Press, Beijing, 2013).

2 Gong, L. & Xu, Q. in *Determination of total acid in foods* Vol. GB/T 12456-2008 (China Agriculture Press, Beijing, 2008).

3 Zhou, W., Xu, J. & Xu, L. in *Determination of total polyphenols and catechins content in tea* Vol. GB/T 8313-2008 (China Agriculture Press, Beijing, 2008).

4 Xu, J., Zhou, W., Lu, X., Wang, J. & Sha, H. in *Tea-Determination of free amino acids content* Vol. GB/T 8314-2013 (China Agriculture Press, Beijing, 2013).

5 Yinhua, L., Juan, L., Xue, G. & Zhonghua, L. Simultaneous determination of eight catechins, three purine alkaloids and gallic acid in tea by high-performance liquid chromatography. *Food Science* **32**, 214-217 (2011).

6 Petry, R. D., Ortega, G. G. & Silva, W. B. Flavonoid content assay: influence of the reagent concentration and reaction time on the spectrophotometric behavior of the aluminium chloride--flavonoid complex. *Die Pharmazie* **56**, 465-470 (2001).

7 Mengxi, L., Zhigang, X., Yang, Y. & Yijie, F. Effects of different spectral lights on Oncidium PLBs induction, proliferation, and plant regeneration. *Plant Cell, Tissue and Organ Culture (PCTOC)* **106**, 1-10 (2011).

.

**Table-S1 The information of identified OTUs during the manufacturing process of Fu brick tea**

|  | **S1** | **S2** | **S3** | **S4** | **S5** | **S6** | **S7** | **S8** | **S9** | **S10** | **taxonomy** |
| --- | --- | --- | --- | --- | --- | --- | --- | --- | --- | --- | --- |
| **OTU1** | 0 | 0 | 11 | 0 | 0 | 0 | 0 | 0 | 0 | 0 | d__Eukaryota; k__Fungi; p__Ascomycota; c__Leotiomycetes; o__Incertae_sedis; f__Myxotrichaceae; g__Pseudogymnoascus; s__Pseudogymnoascus_roseus |
| **OTU2** | 0 | 7 | 0 | 0 | 0 | 0 | 0 | 0 | 0 | 0 | d__Eukaryota; k__Fungi; p__Ascomycota; c__Eurotiomycetes; o__Chaetothyriales; f__Herpotrichiellaceae; g__Exophiala; s__Exophiala_pisciphila |
| **OTU3** | 1 | 1 | 0 | 3 | 0 | 0 | 0 | 0 | 0 | 0 | d__Eukaryota; k__Fungi; p__Ascomycota; c__Eurotiomycetes |
| **OTU4** | 50 | 51 | 57 | 97 | 0 | 0 | 0 | 0 | 0 | 0 | d__Eukaryota; k__Fungi; p__Ascomycota; c__Saccharomycetes; o__Saccharomycetales; f__Incertae_sedis; g__Debaryomyces; s__Debaryomyces_prosopidis |
| **OTU5** | 0 | 1 | 8 | 0 | 0 | 0 | 0 | 0 | 0 | 0 | d__Eukaryota; k__Fungi; p__Zygomycota; c__Incertae_sedis; o__Mortierellales; f__Mortierellaceae; g__Mortierella; s__Mortierella_amoeboidea |
| **OTU6** | 46 | 0 | 3 | 4 | 0 | 0 | 0 | 0 | 0 | 0 | d__Eukaryota; k__Fungi; p__Ascomycota; c__Eurotiomycetes; o__Eurotiales; f__Aspergillaceae; g__Penicillium |
| **OTU7** | 2477 | 2660 | 1570 | 3465 | 1 | 1 | 0 | 0 | 0 | 0 | d__Eukaryota; k__Fungi; p__Ascomycota; c__Eurotiomycetes; o__Eurotiales; f__Aspergillaceae; g__Aspergillus |
| **OTU8** | 0 | 1 | 0 | 0 | 0 | 0 | 0 | 0 | 0 | 0 | d__Eukaryota; k__Fungi; p__Ascomycota; c__Sordariomycetes; o__Hypocreales; f__Hypocreaceae; g__Hypocrea; s__Hypocrea_lactea |
| **OTU9** | 0 | 11 | 1 | 0 | 0 | 0 | 0 | 0 | 0 | 0 | d__Eukaryota; k__Fungi; p__Ascomycota; c__Dothideomycetes; o__Pleosporales; f__Sporormiaceae; g__Preussia; s__Preussia_flanaganii |
| **OTU10** | 1 | 3 | 0 | 1 | 0 | 0 | 0 | 0 | 0 | 0 | d__Eukaryota; k__Fungi; p__Ascomycota; c__Dothideomycetes; o__Capnodiales |
| **OTU11** | 4 | 0 | 6 | 29 | 0 | 0 | 0 | 0 | 0 | 0 | d__Eukaryota; k__Fungi; p__Ascomycota; c__Sordariomycetes; o__Hypocreales; f__Nectriaceae; g__Fusarium; s__Fusarium_delphinoides |
| **OTU12** | 1694 | 1566 | 2891 | 2146 | 0 | 0 | 1 | 0 | 1 | 1 | d__Eukaryota; k__Fungi; p__Ascomycota; c__Eurotiomycetes; o__Eurotiales; f__Aspergillaceae; g__Aspergillus |
| **OTU13** | 0 | 0 | 6 | 0 | 0 | 0 | 0 | 0 | 0 | 0 | d__Eukaryota; k__Fungi; p__Ascomycota; c__Pezizomycetes; o__Pezizales; f__Pyronemataceae |
| **OTU14** | 0 | 2 | 0 | 0 | 0 | 0 | 0 | 0 | 0 | 0 | d__Eukaryota; k__Fungi; p__Ascomycota; c__Incertae_sedis; o__Incertae_sedis; f__Pseudeurotiaceae; g__unidentified; s__Pseudeurotiaceae_sp |
| **OTU15** | 0 | 0 | 3 | 0 | 0 | 0 | 0 | 0 | 0 | 0 | d__Eukaryota; k__Fungi; p__Basidiomycota; c__Tremellomycetes; o__Tremellales; f__Incertae_sedis; g__Cryptococcus; s__Cryptococcus_magnus |
| **OTU16** | 0 | 7 | 0 | 0 | 0 | 0 | 0 | 0 | 0 | 0 | d__Eukaryota; k__Fungi; p__Ascomycota; c__Dothideomycetes; o__Pleosporales; f__Cucurbitariaceae; g__Pyrenochaetopsis; s__Pyrenochaetopsis_leptospora |
| **OTU17** | 1 | 0 | 3 | 6 | 0 | 0 | 0 | 0 | 0 | 0 | d__Eukaryota; k__Fungi; p__Ascomycota; c__Saccharomycetes; o__Saccharomycetales; f__Trichomonascaceae; g__Blastobotrys; s__Blastobotrys_adeninivorans |
| **OTU18** | 2 | 0 | 0 | 0 | 0 | 0 | 0 | 0 | 0 | 0 | d__Eukaryota; k__Fungi; p__Ascomycota; c__Sordariomycetes; o__Ophiostomatales; f__Ophiostomataceae; g__Sporothrix; s__Sporothrix_stylites |
| **OTU19** | 3 | 1 | 3 | 14 | 0 | 0 | 0 | 0 | 0 | 0 | d__Eukaryota; k__Fungi; p__Ascomycota; c__Eurotiomycetes; o__Eurotiales; f__Aspergillaceae; g__Penicillium; s__Penicillium_sp |
| **OTU20** | 0 | 0 | 5 | 0 | 0 | 0 | 0 | 0 | 0 | 0 | d__Eukaryota; k__Fungi; p__Ascomycota; c__Dothideomycetes; o__Capnodiales; f__Davidiellaceae; g__Cladosporium; s__Cladosporium_sphaerospermum |
| **OTU21** | 0 | 4 | 0 | 0 | 0 | 0 | 0 | 0 | 0 | 0 | d__Eukaryota; k__Fungi; p__Ascomycota; c__Sordariomycetes; o__Sordariales; f__Lasiosphaeriaceae; g__unidentified; s__Lasiosphaeriaceae_sp |
| **OTU22** | 3 | 6 | 10 | 12 | 0 | 0 | 0 | 0 | 0 | 0 | d__Eukaryota; k__Fungi; p__Ascomycota; c__Leotiomycetes; o__Helotiales; f__Sclerotiniaceae |
| **OTU23** | 1 | 0 | 0 | 1 | 0 | 0 | 0 | 0 | 0 | 0 | d__Eukaryota; k__Fungi; p__Ascomycota |
| **OTU24** | 2 | 13 | 38 | 10 | 0 | 0 | 0 | 0 | 0 | 0 | d__Eukaryota; k__Fungi |
| **OTU25** | 0 | 11 | 3 | 0 | 0 | 0 | 0 | 0 | 0 | 1 | d__Eukaryota; k__Fungi; p__Ascomycota; c__Sordariomycetes; o__Hypocreales; f__Nectriaceae; g__Gibberella; s__Gibberella_intricans |
| **OTU26** | 228 | 1 | 0 | 0 | 0 | 0 | 0 | 0 | 0 | 0 | d__Eukaryota; k__Fungi; p__Ascomycota; c__Sordariomycetes; o__Incertae_sedis; f__Incertae_sedis; g__Eucasphaeria; s__Eucasphaeria_capensis |
| **OTU27** | 0 | 2 | 0 | 0 | 0 | 0 | 0 | 0 | 0 | 0 | d__Eukaryota; k__Fungi; p__Ascomycota; c__Sordariomycetes; o__Hypocreales |
| **OTU28** | 0 | 0 | 3 | 0 | 0 | 0 | 0 | 0 | 0 | 0 | d__Eukaryota; k__Fungi; p__Ascomycota; c__Eurotiomycetes; o__Eurotiales; f__Aspergillaceae |
| **OTU29** | 0 | 10 | 4 | 2 | 0 | 0 | 0 | 0 | 0 | 0 | d__Eukaryota; k__Fungi; p__Ascomycota; c__Eurotiomycetes; o__Eurotiales; f__Aspergillaceae; g__Aspergillus; s__Aspergillus_flavus |
| **OTU30** | 16 | 2 | 19 | 14 | 0 | 0 | 0 | 0 | 0 | 0 | d__Eukaryota; k__Fungi; p__Ascomycota; c__Sordariomycetes; o__Hypocreales |
| **OTU31** | 0 | 5 | 4 | 0 | 0 | 0 | 0 | 0 | 0 | 0 | d__Eukaryota; k__Fungi; p__Ascomycota; c__Sordariomycetes; o__Hypocreales; f__Hypocreaceae |
| **OTU32** | 0 | 0 | 1 | 0 | 0 | 0 | 0 | 0 | 0 | 0 | d__Eukaryota; k__Fungi; p__Ascomycota; c__Sordariomycetes; o__Hypocreales; f__Cordycipitaceae; g__Lecanicillium; s__Lecanicillium_fusisporum |
| **OTU33** | 53 | 0 | 8 | 6 | 0 | 0 | 0 | 0 | 0 | 0 | d__Eukaryota; k__Fungi; p__Ascomycota; c__Dothideomycetes; o__Capnodiales; f__Mycosphaerellaceae; g__Uwebraunia; s__Uwebraunia_musae |
| **OTU34** | 1 | 0 | 0 | 2 | 0 | 0 | 0 | 0 | 0 | 0 | d__Eukaryota; k__Fungi; p__Ascomycota; c__Eurotiomycetes; o__Eurotiales; f__Aspergillaceae; g__Talaromyces; s__Talaromyces_rotundus |
| **OTU35** | 14 | 1 | 1 | 1 | 0 | 0 | 0 | 0 | 0 | 0 | d__Eukaryota; k__Fungi; p__Ascomycota |
| **OTU36** | 4 | 0 | 0 | 0 | 0 | 0 | 0 | 0 | 0 | 0 | d__Eukaryota; k__Fungi; p__Ascomycota; c__Leotiomycetes |
| **OTU37** | 0 | 1 | 0 | 0 | 0 | 0 | 0 | 0 | 0 | 0 | d__Eukaryota; k__Fungi; p__Ascomycota; c__Dothideomycetes; o__Capnodiales |
| **OTU38** | 0 | 4 | 0 | 0 | 0 | 0 | 0 | 0 | 0 | 0 | d__Eukaryota; k__Fungi; p__Ascomycota; c__Dothideomycetes; o__Pleosporales; f__Sporormiaceae; g__Preussia |
| **OTU39** | 347 | 27 | 73 | 34 | 0 | 0 | 0 | 0 | 0 | 0 | d__Eukaryota; k__Fungi; p__Ascomycota; c__Eurotiomycetes; o__Eurotiales; f__Aspergillaceae; g__Penicillium; s__Penicillium_citrinum |
| **OTU40** | 5 | 0 | 0 | 0 | 0 | 0 | 0 | 0 | 0 | 0 | d__Eukaryota; k__Fungi; p__Ascomycota; c__Sordariomycetes; o__Hypocreales; f__Bionectriaceae; g__unidentified; s__Bionectriaceae_sp |
| **OTU41** | 0 | 5 | 0 | 0 | 0 | 0 | 0 | 0 | 0 | 0 | d__Eukaryota; k__Fungi; p__Ascomycota; c__Sordariomycetes; o__Hypocreales; f__Incertae_sedis; g__Stachybotrys; s__Stachybotrys_eucylindrospora |
| **OTU42** | 3 | 22 | 20 | 9 | 0 | 0 | 0 | 0 | 0 | 0 | d__Eukaryota; k__Fungi; p__Basidiomycota; c__Tremellomycetes; o__Trichosporonales; f__Trichosporonaceae; g__Trichosporon; s__Trichosporon_asahii |
| **OTU43** | 1 | 0 | 0 | 0 | 0 | 0 | 0 | 0 | 0 | 0 | d__Eukaryota; k__Fungi; p__Ascomycota; c__Sordariomycetes; o__Microascales; f__Microascaceae; g__Microascus |
| **OTU44** | 1 | 0 | 9 | 3 | 0 | 0 | 0 | 0 | 0 | 0 | d__Eukaryota; k__Fungi; p__Ascomycota; c__Dothideomycetes; o__Incertae_sedis; f__Eremomycetaceae; g__Arthrographis; s__Arthrographis_sp |
| **OTU45** | 169 | 66 | 604 | 342 | 0 | 0 | 0 | 0 | 0 | 0 | d__Eukaryota; k__Fungi; p__Basidiomycota; c__Wallemiomycetes; o__Wallemiales; f__Wallemiaceae; g__Wallemia; s__Wallemia_sebi |
| **OTU46** | 0 | 0 | 2 | 3 | 0 | 0 | 0 | 0 | 0 | 0 | d__Eukaryota; k__Fungi; p__Ascomycota; c__Saccharomycetes; o__Saccharomycetales; f__Incertae_sedis; g__Cyberlindnera; s__Cyberlindnera_jadinii |
| **OTU47** | 0 | 0 | 0 | 3 | 0 | 0 | 0 | 0 | 0 | 0 | d__Eukaryota; k__Fungi; p__Ascomycota; c__Sordariomycetes; o__Hypocreales; f__Incertae_sedis; g__Acremonium; s__Acremonium_sp |
| **OTU48** | 0 | 2 | 1 | 0 | 0 | 0 | 0 | 0 | 0 | 0 | d__Eukaryota; k__Fungi; p__Ascomycota; c__Eurotiomycetes; o__Eurotiales; f__Aspergillaceae; g__Penicillium; s__Penicillium_jugoslavicum |
| **OTU49** | 0 | 1 | 0 | 0 | 0 | 0 | 0 | 0 | 0 | 0 | d__Eukaryota; k__Fungi; p__Ascomycota; c__Sordariomycetes; o__Sordariales |
| **OTU50** | 2 | 2 | 20 | 5 | 0 | 0 | 0 | 0 | 0 | 0 | d__Eukaryota; k__Fungi; p__Zygomycota; c__Incertae_sedis; o__Mucorales; f__Lichtheimiaceae; g__Rhizomucor; s__Rhizomucor_pusillus |
| **OTU51** | 0 | 0 | 6 | 0 | 0 | 0 | 0 | 0 | 0 | 0 | d__Eukaryota; k__Fungi; p__Ascomycota; c__Dothideomycetes; o__Capnodiales |
| **OTU52** | 10 | 75 | 33 | 24 | 0 | 0 | 0 | 1 | 0 | 0 | d__Eukaryota; k__Fungi; p__Ascomycota; c__Dothideomycetes; o__Capnodiales; f__Davidiellaceae; g__Cladosporium; s__Cladosporium_ramotenellum |
| **OTU53** | 6 | 74 | 51 | 17 | 0 | 0 | 0 | 0 | 0 | 0 | d__Eukaryota; k__Fungi; p__Ascomycota; c__Dothideomycetes; o__Pleosporales; f__Incertae_sedis; g__Phoma |
| **OTU54** | 0 | 4 | 0 | 0 | 0 | 0 | 0 | 0 | 0 | 0 | d__Eukaryota; k__Fungi; p__Ascomycota; c__Sordariomycetes; o__Hypocreales; f__Incertae_sedis; g__Myrothecium |
| **OTU55** | 62 | 0 | 0 | 0 | 0 | 0 | 0 | 0 | 0 | 0 | d__Eukaryota; k__Fungi; p__Ascomycota; c__Eurotiomycetes; o__Eurotiales; f__Aspergillaceae; g__Aspergillus; s__Aspergillus_ustus |
| **OTU56** | 0 | 0 | 0 | 5 | 0 | 0 | 0 | 0 | 0 | 0 | d__Eukaryota; k__Fungi; p__Ascomycota |
| **OTU57** | 0 | 0 | 2 | 0 | 0 | 0 | 0 | 0 | 0 | 0 | d__Eukaryota; k__Fungi; p__Ascomycota; c__Sordariomycetes; o__Glomerellales; f__Glomerellaceae; g__Colletotrichum |
| **OTU58** | 91 | 346 | 160 | 39 | 0 | 0 | 0 | 0 | 0 | 0 | d__Eukaryota; k__Fungi; p__Ascomycota; c__Eurotiomycetes; o__Eurotiales; f__Aspergillaceae; g__Aspergillus |
| **OTU59** | 0 | 2 | 0 | 0 | 0 | 0 | 0 | 0 | 0 | 0 | d__Eukaryota; k__Fungi |
| **OTU60** | 0 | 3 | 1 | 0 | 0 | 0 | 0 | 0 | 0 | 0 | d__Eukaryota; k__Fungi; p__Ascomycota; c__Dothideomycetes; o__Dothideales; f__Dothioraceae; g__Selenophoma; s__Selenophoma_mahoniae |
| **OTU61** | 0 | 2 | 1 | 0 | 0 | 0 | 0 | 0 | 0 | 0 | d__Eukaryota; k__Fungi; p__Ascomycota; c__Sordariomycetes; o__Xylariales; f__Xylariaceae; g__Ascotricha; s__Ascotricha_sp_OUCMB0118 |
| **OTU62** | 0 | 0 | 0 | 16 | 0 | 0 | 0 | 0 | 0 | 0 | d__Eukaryota; k__Fungi; p__Basidiomycota; c__Microbotryomycetes; o__Sporidiobolales; f__Incertae_sedis |
| **OTU63** | 38 | 0 | 0 | 0 | 0 | 0 | 0 | 0 | 0 | 0 | d__Eukaryota; k__Fungi; p__Ascomycota |
| **OTU64** | 0 | 12 | 0 | 0 | 0 | 0 | 0 | 0 | 0 | 0 | d__Eukaryota; k__Fungi; p__Ascomycota; c__Sordariomycetes; o__Incertae_sedis; f__Incertae_sedis; g__Myrmecridium; s__Myrmecridium_sp |
| **OTU65** | 3 | 0 | 1 | 2 | 0 | 0 | 0 | 0 | 0 | 0 | d__Eukaryota; k__Fungi; p__Ascomycota; c__Eurotiomycetes; o__Eurotiales |
| **OTU66** | 1 | 0 | 5 | 0 | 0 | 0 | 0 | 0 | 0 | 0 | d__Eukaryota; k__Fungi; p__Ascomycota; c__Sordariomycetes; o__Sordariales; f__Chaetomiaceae |
| **OTU67** | 0 | 0 | 4 | 0 | 0 | 0 | 0 | 0 | 0 | 0 | d__Eukaryota; k__Fungi; p__Ascomycota |
| **OTU68** | 78 | 336 | 468 | 134 | 0 | 0 | 0 | 0 | 0 | 0 | d__Eukaryota; k__Fungi; p__Ascomycota; c__Eurotiomycetes; o__Eurotiales; f__Aspergillaceae; g__Aspergillus; s__Aspergillus_sp_F55 |
| **OTU69** | 0 | 2 | 4 | 2 | 0 | 0 | 0 | 0 | 0 | 0 | d__Eukaryota; k__Fungi; p__Basidiomycota; c__Tremellomycetes; o__Tremellales; f__Incertae_sedis; g__Cryptococcus; s__Cryptococcus_fragicola |
| **OTU70** | 0 | 1 | 2 | 0 | 0 | 0 | 0 | 0 | 0 | 0 | d__Eukaryota; k__Fungi; p__Ascomycota; c__Eurotiomycetes; o__Chaetothyriales; f__Incertae_sedis; g__Strelitziana |
| **OTU71** | 37 | 13 | 52 | 29 | 0 | 0 | 0 | 0 | 0 | 3 | d__Eukaryota; k__Fungi; p__Ascomycota; c__Sordariomycetes |
| **OTU72** | 1918 | 9448 | 2425 | 1806 | 20826 | 20806 | 20824 | 20819 | 20817 | 20787 | d__Eukaryota; k__Fungi; p__Ascomycota; c__Eurotiomycetes; o__Eurotiales; f__Aspergillaceae; g__Aspergillus; s__Aspergillus_ cristatus |
| **OTU73** | 0 | 0 | 3 | 1 | 0 | 0 | 0 | 0 | 0 | 0 | d__Eukaryota; k__Fungi; p__Ascomycota |
| **OTU74** | 32 | 0 | 36 | 5 | 0 | 0 | 0 | 0 | 0 | 2 | d__Eukaryota; k__Fungi; p__Ascomycota; c__Eurotiomycetes; o__Eurotiales; f__Aspergillaceae; g__Penicillium; s__Penicillium_brocae |
| **OTU75** | 16 | 7 | 25 | 8 | 0 | 0 | 0 | 0 | 0 | 0 | d__Eukaryota; k__Fungi; p__Ascomycota; c__Dothideomycetes; o__Pleosporales; f__unidentified; g__unidentified; s__Pleosporales_sp |
| **OTU76** | 8 | 0 | 0 | 0 | 0 | 0 | 0 | 0 | 0 | 0 | d__Eukaryota; k__Fungi; p__Ascomycota |
| **OTU77** | 16 | 0 | 0 | 0 | 0 | 0 | 0 | 0 | 0 | 0 | d__Eukaryota; k__Fungi; p__Ascomycota; c__Eurotiomycetes; o__Eurotiales; f__Aspergillaceae; g__Penicillium; s__Penicillium_implicatum |
| **OTU78** | 0 | 1 | 5 | 0 | 0 | 0 | 0 | 0 | 0 | 0 | d__Eukaryota; k__Fungi; p__Ascomycota; c__Lecanoromycetes; o__Lecanorales; f__Ramalinaceae |
| **OTU79** | 0 | 0 | 3 | 1 | 0 | 0 | 0 | 0 | 0 | 0 | d__Eukaryota; k__Fungi; p__Ascomycota; c__Dothideomycetes; o__Botryosphaeriales; f__Botryosphaeriaceae; g__Microdiplodia; s__Microdiplodia_sp |
| **OTU80** | 4 | 0 | 0 | 0 | 0 | 0 | 0 | 0 | 0 | 0 | d__Eukaryota; k__Fungi; p__Ascomycota; c__Sordariomycetes; o__Glomerellales; f__Glomerellaceae; g__Glomerella; s__Glomerella_cingulata |
| **OTU81** | 3 | 8 | 43 | 0 | 0 | 0 | 0 | 0 | 0 | 0 | d__Eukaryota; k__Fungi; p__Ascomycota; c__Sordariomycetes; o__Hypocreales; f__Nectriaceae; g__Fusarium |
| **OTU82** | 0 | 2 | 0 | 0 | 0 | 0 | 0 | 0 | 0 | 0 | d__Eukaryota; k__Fungi; p__Ascomycota; c__Eurotiomycetes |
| **OTU83** | 1 | 3 | 4 | 0 | 0 | 0 | 0 | 0 | 0 | 0 | d__Eukaryota; k__Fungi; p__Ascomycota; c__Dothideomycetes; o__Pleosporales; f__Incertae_sedis |
| **OTU84** | 0 | 2 | 2 | 0 | 0 | 0 | 0 | 0 | 0 | 0 | d__Eukaryota; k__Fungi; p__Basidiomycota; c__Tremellomycetes; o__Cystofilobasidiales; f__Cystofilobasidiaceae; g__Mrakia; s__Mrakia_frigida |
| **OTU85** | 0 | 0 | 2 | 0 | 0 | 0 | 0 | 0 | 0 | 0 | d__Eukaryota; k__Fungi; p__Ascomycota; c__Sordariomycetes; o__Hypocreales; f__Cordycipitaceae; g__Cordyceps; s__Cordyceps_bassiana |
| **OTU86** | 42 | 76 | 256 | 71 | 0 | 0 | 0 | 0 | 0 | 0 | d__Eukaryota; k__Fungi |
| **OTU87** | 0 | 2 | 0 | 0 | 0 | 0 | 0 | 0 | 0 | 0 | d__Eukaryota; k__Fungi; p__Basidiomycota; c__Agaricomycetes; o__Auriculariales; f__Incertae_sedis; g__Heterochaete; s__Heterochaete_shearii |
| **OTU88** | 12 | 3 | 15 | 1 | 0 | 0 | 0 | 0 | 0 | 0 | d__Eukaryota; k__Fungi; p__Ascomycota; c__Dothideomycetes; o__Capnodiales; f__Incertae_sedis; g__Rachicladosporium; s__Rachicladosporium_sp_HX6 |
| **OTU89** | 0 | 4 | 0 | 0 | 0 | 0 | 0 | 0 | 0 | 0 | d__Eukaryota; k__Fungi; p__Ascomycota; c__Dothideomycetes; o__Capnodiales; f__Mycosphaerellaceae |
| **OTU90** | 0 | 0 | 2 | 0 | 0 | 0 | 0 | 0 | 0 | 0 | d__Eukaryota; k__Fungi; p__Ascomycota; c__Sordariomycetes; o__Sordariales; f__Lasiosphaeriaceae; g__Cercophora; s__Cercophora_sp |
| **OTU91** | 1 | 3 | 9 | 2 | 0 | 0 | 0 | 0 | 0 | 0 | d__Eukaryota; k__Fungi; p__Ascomycota; c__Eurotiomycetes; o__Chaetothyriales; f__Incertae_sedis; g__Strelitziana |
| **OTU92** | 1 | 0 | 0 | 0 | 0 | 0 | 0 | 0 | 0 | 0 | d__Eukaryota; k__Fungi; p__Ascomycota; c__Sordariomycetes; o__Microascales; f__Microascaceae |
| **OTU93** | 4 | 1 | 2 | 0 | 0 | 0 | 0 | 0 | 0 | 0 | d__Eukaryota; k__Fungi; p__Ascomycota; c__Dothideomycetes; o__Myriangiales; f__Elsinoaceae; g__Elsinoe; s__Elsinoe_fawcettii |
| **OTU94** | 1 | 4 | 0 | 1 | 0 | 0 | 0 | 0 | 0 | 0 | d__Eukaryota; k__Fungi; p__Ascomycota; c__Dothideomycetes; o__Capnodiales; f__Mycosphaerellaceae |
| **OTU95** | 2 | 0 | 1 | 0 | 0 | 0 | 0 | 0 | 0 | 0 | d__Eukaryota; k__Fungi; p__Ascomycota; c__Eurotiomycetes; o__Eurotiales; f__Aspergillaceae; g__Byssochlamys |
| **OTU96** | 0 | 0 | 3 | 2 | 0 | 0 | 0 | 0 | 0 | 0 | d__Eukaryota; k__Fungi; p__Ascomycota; c__Eurotiomycetes; o__Chaetothyriales; f__Herpotrichiellaceae; g__Phialophora; s__Phialophora_sessilis |
| **OTU97** | 4 | 1 | 1 | 2 | 0 | 0 | 0 | 0 | 0 | 0 | d__Eukaryota; k__Fungi; p__Ascomycota; c__Sordariomycetes; o__Hypocreales |
| **OTU98** | 0 | 4 | 3 | 0 | 0 | 0 | 0 | 0 | 0 | 0 | d__Eukaryota; k__Fungi; p__Ascomycota; c__Dothideomycetes; o__Incertae_sedis; f__Eremomycetaceae; g__Arthrographis; s__Arthrographis_curvata |
| **OTU99** | 0 | 1 | 0 | 0 | 0 | 0 | 0 | 0 | 0 | 0 | d__Eukaryota; k__Fungi; p__Ascomycota; c__Sordariomycetes; o__Hypocreales; f__Incertae_sedis; g__Stachybotrys; s__Stachybotrys_kampalensis |
| **OTU100** | 1422 | 3137 | 899 | 341 | 0 | 0 | 0 | 0 | 0 | 0 | d__Eukaryota; k__Fungi; p__Ascomycota; c__Eurotiomycetes; o__Eurotiales; f__Aspergillaceae; g__Aspergillus; s__Aspergillus_penicillioides |
| **OTU101** | 0 | 9 | 0 | 1 | 0 | 0 | 0 | 0 | 0 | 0 | d__Eukaryota; k__Fungi; p__Basidiomycota; c__Microbotryomycetes; o__Sporidiobolales; f__Incertae_sedis; g__Rhodotorula |
| **OTU102** | 0 | 4 | 0 | 0 | 0 | 0 | 0 | 0 | 0 | 0 | d__Eukaryota; k__Fungi |
| **OTU103** | 0 | 2 | 0 | 0 | 0 | 0 | 0 | 0 | 0 | 0 | d__Eukaryota; k__Fungi; p__Basidiomycota; c__Microbotryomycetes; o__Sporidiobolales; f__Incertae_sedis; g__Rhodotorula; s__Rhodotorula_mucilaginosa |
| **OTU104** | 2 | 0 | 20 | 30 | 0 | 0 | 0 | 0 | 0 | 0 | d__Eukaryota; k__Fungi; p__Ascomycota; c__Saccharomycetes; o__Saccharomycetales |
| **OTU105** | 0 | 0 | 0 | 0 | 0 | 0 | 0 | 0 | 0 | 2 | d__Eukaryota; k__Fungi; p__Zygomycota; c__Incertae_sedis; o__Mortierellales; f__Mortierellaceae; g__Mortierella |
| **OTU106** | 0 | 8 | 0 | 0 | 0 | 0 | 0 | 0 | 0 | 0 | d__Eukaryota; k__Fungi; p__Zygomycota; c__Incertae_sedis; o__Mortierellales; f__Mortierellaceae; g__Mortierella; s__Mortierella_polygonia |
| **OTU107** | 0 | 3 | 0 | 0 | 0 | 0 | 0 | 0 | 0 | 0 | d__Eukaryota; k__Fungi; p__Ascomycota; c__Eurotiomycetes |
| **OTU108** | 4887 | 306 | 2073 | 5331 | 0 | 0 | 0 | 0 | 1 | 9 | d__Eukaryota; k__Fungi; p__Ascomycota; c__Saccharomycetes; o__Saccharomycetales |
| **OTU109** | 920 | 69 | 74 | 236 | 0 | 0 | 0 | 0 | 0 | 0 | d__Eukaryota; k__Fungi; p__Ascomycota; c__Eurotiomycetes; o__Eurotiales; f__Aspergillaceae; g__Aspergillus |
| **OTU110** | 0 | 0 | 8 | 0 | 0 | 0 | 0 | 0 | 0 | 0 | d__Eukaryota; k__Fungi |
| **OTU111** | 2 | 1 | 0 | 1 | 0 | 0 | 0 | 0 | 0 | 0 | d__Eukaryota; k__Fungi; p__Ascomycota; c__Sordariomycetes; o__Sordariales |
| **OTU112** | 0 | 9 | 0 | 0 | 0 | 0 | 0 | 0 | 0 | 0 | d__Eukaryota; k__Fungi; p__Ascomycota; c__Sordariomycetes; o__Sordariales; f__Lasiosphaeriaceae; g__Podospora; s__Podospora_sp |
| **OTU113** | 1 | 2 | 0 | 0 | 0 | 0 | 0 | 0 | 0 | 0 | d__Eukaryota; k__Fungi; p__Ascomycota; c__Dothideomycetes; o__Capnodiales; f__Mycosphaerellaceae; g__Pseudocercosporella; s__Pseudocercosporella_sp |
| **OTU114** | 205 | 35 | 155 | 19 | 0 | 0 | 0 | 0 | 0 | 0 | d__Eukaryota; k__Fungi; p__Ascomycota; c__Eurotiomycetes; o__Eurotiales; f__Aspergillaceae; g__Aspergillus; s__Aspergillus_sp_NRRL_145 |
| **OTU115** | 0 | 14 | 9 | 5 | 0 | 0 | 0 | 0 | 0 | 0 | d__Eukaryota; k__Fungi; p__Basidiomycota; c__Tremellomycetes; o__Cystofilobasidiales; f__Cystofilobasidiaceae; g__Guehomyces; s__Guehomyces_pullulans |
| **OTU116** | 0 | 1 | 0 | 0 | 0 | 0 | 0 | 0 | 0 | 0 | d__Eukaryota; k__Fungi; p__Ascomycota |
| **OTU117** | 0 | 1 | 1 | 0 | 0 | 0 | 0 | 0 | 0 | 0 | d__Eukaryota; k__Fungi; p__Ascomycota; c__Saccharomycetes; o__Saccharomycetales; f__Incertae_sedis; g__Candida; s__Candida_blankii |
| **OTU118** | 1 | 0 | 0 | 1 | 0 | 0 | 0 | 0 | 0 | 0 | d__Eukaryota; k__Fungi; p__Ascomycota; c__Dothideomycetes; o__Capnodiales; f__Mycosphaerellaceae |
| **OTU119** | 1 | 0 | 7 | 4 | 0 | 0 | 0 | 0 | 0 | 0 | d__Eukaryota; k__Fungi; p__Ascomycota; c__Sordariomycetes |
| **OTU120** | 0 | 0 | 0 | 1 | 0 | 0 | 0 | 0 | 0 | 0 | d__Eukaryota; k__Fungi; p__Ascomycota; c__Eurotiomycetes; o__Eurotiales; f__Aspergillaceae; g__Sagenomella; s__Sagenomella_sp |
| **OTU121** | 0 | 5 | 7 | 0 | 0 | 0 | 0 | 0 | 0 | 0 | d__Eukaryota; k__Fungi; p__Ascomycota; c__Dothideomycetes; o__Pleosporales; f__Sporormiaceae |
| **OTU122** | 0 | 4 | 10 | 129 | 0 | 0 | 0 | 0 | 0 | 0 | d__Eukaryota; k__Fungi; p__Basidiomycota; c__Tremellomycetes; o__Trichosporonales; f__Trichosporonaceae; g__Trichosporon |
| **OTU123** | 0 | 14 | 0 | 0 | 0 | 0 | 0 | 0 | 0 | 0 | d__Eukaryota; k__Fungi; p__Ascomycota; c__Sordariomycetes |
| **OTU124** | 0 | 0 | 2 | 0 | 0 | 0 | 0 | 0 | 0 | 0 | d__Eukaryota; k__Fungi; p__Basidiomycota; c__Microbotryomycetes; o__Sporidiobolales |
| **OTU125** | 0 | 0 | 2 | 0 | 0 | 0 | 0 | 0 | 0 | 0 | d__Eukaryota; k__Fungi; p__Ascomycota |
| **OTU126** | 0 | 0 | 7 | 0 | 0 | 0 | 0 | 0 | 0 | 0 | d__Eukaryota; k__Fungi; p__Basidiomycota; c__Tremellomycetes; o__Cystofilobasidiales; f__Cystofilobasidiaceae; g__Itersonilia; s__Itersonilia_perplexans |
| **OTU127** | 1 | 0 | 0 | 1 | 0 | 0 | 0 | 0 | 0 | 0 | d__Eukaryota; k__Fungi; p__Ascomycota; c__Dothideomycetes; o__Capnodiales |
| **OTU128** | 4 | 11 | 13 | 3 | 0 | 0 | 0 | 0 | 0 | 1 | d__Eukaryota; k__Fungi; p__Ascomycota; c__Dothideomycetes; o__Pleosporales; f__Pleosporaceae; g__Alternaria |
| **OTU129** | 0 | 5 | 0 | 0 | 0 | 0 | 0 | 0 | 0 | 0 | d__Eukaryota; k__Fungi; p__Ascomycota; c__Dothideomycetes; o__Pleosporales; f__Phaeosphaeriaceae; g__Sclerostagonospora; s__Sclerostagonospora_sp |
| **OTU130** | 0 | 5 | 0 | 0 | 0 | 0 | 0 | 0 | 0 | 0 | d__Eukaryota; k__Fungi; p__Ascomycota |
| **OTU131** | 0 | 4 | 0 | 0 | 0 | 0 | 0 | 0 | 0 | 0 | d__Eukaryota; k__Fungi; p__Basidiomycota; c__Agaricomycetes; o__Agaricales; f__Schizophyllaceae; g__Schizophyllum; s__Schizophyllum_commune |
| **OTU132** | 0 | 9 | 5 | 5 | 0 | 0 | 0 | 0 | 0 | 0 | d__Eukaryota; k__Fungi; p__Ascomycota; c__Dothideomycetes; o__Pleosporales; f__Pleosporaceae; g__Epicoccum; s__Epicoccum_nigrum |
| **OTU133** | 0 | 5 | 0 | 0 | 0 | 0 | 0 | 0 | 0 | 0 | d__Eukaryota; k__Fungi; p__Ascomycota; c__Dothideomycetes; o__Pleosporales; f__Pleosporaceae; g__Setosphaeria; s__Setosphaeria_turcica |
| **OTU134** | 0 | 0 | 24 | 0 | 0 | 0 | 0 | 0 | 0 | 0 | d__Eukaryota; k__Fungi; p__Ascomycota; c__Sordariomycetes; o__Hypocreales; f__Nectriaceae |
| **OTU135** | 67 | 18 | 3253 | 825 | 1 | 0 | 0 | 2 | 0 | 2 | d__Eukaryota; k__Fungi; p__Ascomycota; c__Saccharomycetes; o__Saccharomycetales; f__Incertae_sedis; g__Candida; s__Candida_metapsilosis |
| **OTU136** | 2 | 0 | 217 | 68 | 0 | 0 | 0 | 0 | 0 | 0 | d__Eukaryota; k__Fungi; p__Ascomycota; c__Eurotiomycetes; o__Eurotiales; f__Aspergillaceae |
| **OTU137** | 0 | 0 | 3 | 0 | 0 | 0 | 0 | 0 | 0 | 0 | d__Eukaryota; k__Fungi; p__Ascomycota; c__Eurotiomycetes; o__Eurotiales |
| **OTU138** | 4 | 0 | 0 | 0 | 0 | 0 | 0 | 0 | 0 | 0 | d__Eukaryota; k__Fungi; p__Ascomycota; c__Eurotiomycetes; o__Eurotiales; f__Aspergillaceae; g__Penicillium |
| **OTU139** | 5 | 7 | 1 | 7 | 0 | 0 | 0 | 0 | 0 | 0 | d__Eukaryota; k__Fungi; p__Ascomycota; c__Dothideomycetes; o__Pleosporales; f__Montagnulaceae; g__Paraconiothyrium |
| **OTU140** | 7 | 8 | 34 | 1 | 0 | 0 | 0 | 0 | 0 | 0 | d__Eukaryota; k__Fungi; p__Ascomycota; c__Eurotiomycetes; o__Eurotiales; f__Aspergillaceae; g__Penicillium |
| **OTU141** | 0 | 0 | 3 | 0 | 0 | 0 | 0 | 0 | 0 | 0 | d__Eukaryota; k__Fungi; p__Ascomycota; c__Sordariomycetes |
| **OTU142** | 0 | 1 | 0 | 0 | 0 | 0 | 0 | 0 | 0 | 0 | d__Eukaryota; k__Fungi; p__Ascomycota |
| **OTU143** | 3 | 29 | 9 | 2 | 0 | 0 | 0 | 0 | 0 | 0 | d__Eukaryota; k__Fungi; p__Ascomycota; c__Eurotiomycetes; o__Chaetothyriales; f__Chaetothyriaceae; g__unidentified; s__Chaetothyriaceae_sp |
| **OTU144** | 0 | 2 | 2 | 0 | 0 | 0 | 0 | 0 | 0 | 0 | d__Eukaryota; k__Fungi; p__Ascomycota; c__Sordariomycetes; o__Trichosphaeriales; f__Incertae_sedis; g__unidentified; s__Trichosphaeriales_sp |
| **OTU145** | 241 | 8 | 26 | 253 | 0 | 0 | 0 | 0 | 0 | 0 | d__Eukaryota; k__Fungi; p__Ascomycota; c__Eurotiomycetes; o__Eurotiales; f__Aspergillaceae; g__Aspergillus; s__Aspergillus_fumigatus |
| **OTU146** | 0 | 7 | 0 | 0 | 0 | 0 | 0 | 0 | 0 | 0 | d__Eukaryota; k__Fungi; p__Ascomycota; c__Sordariomycetes; o__Sordariales; f__Lasiosphaeriaceae; g__unidentified; s__Lasiosphaeriaceae_sp |
| **OTU147** | 0 | 3 | 0 | 0 | 0 | 0 | 0 | 0 | 0 | 0 | d__Eukaryota; k__Fungi; p__Ascomycota |
| **OTU148** | 0 | 0 | 0 | 1 | 0 | 0 | 0 | 0 | 0 | 0 | d__Eukaryota; k__Fungi; p__Ascomycota; c__Dothideomycetes; o__Capnodiales |
| **OTU149** | 0 | 22 | 6 | 0 | 0 | 0 | 0 | 0 | 0 | 0 | d__Eukaryota; k__Fungi; p__Zygomycota; c__Incertae_sedis; o__Mortierellales; f__Mortierellaceae; g__Mortierella |
| **OTU150** | 2 | 2 | 14 | 5 | 0 | 0 | 0 | 0 | 0 | 0 | d__Eukaryota; k__Fungi; p__Ascomycota; c__Sordariomycetes; o__Hypocreales |
| **OTU151** | 0 | 0 | 4 | 0 | 0 | 0 | 0 | 0 | 0 | 0 | d__Eukaryota; k__Fungi; p__Basidiomycota; c__Microbotryomycetes; o__Sporidiobolales; f__Incertae_sedis; g__Sporobolomyces; s__Sporobolomyces_oryzicola |
| **OTU152** | 0 | 0 | 6 | 2 | 0 | 0 | 0 | 0 | 0 | 0 | d__Eukaryota; k__Fungi; p__Ascomycota; c__Eurotiomycetes; o__Chaetothyriales; f__Incertae_sedis; g__Strelitziana; s__Strelitziana_mali |
| **OTU153** | 1 | 1 | 0 | 0 | 0 | 0 | 0 | 0 | 0 | 0 | d__Eukaryota; k__Fungi; p__Ascomycota; c__Dothideomycetes |
| **OTU154** | 8 | 33 | 27 | 3 | 0 | 0 | 0 | 0 | 0 | 0 | d__Eukaryota; k__Fungi; p__Ascomycota; c__Eurotiomycetes; o__Eurotiales; f__Aspergillaceae; g__Aspergillus; s__Aspergillus_sp |
| **OTU155** | 5 | 0 | 1 | 0 | 0 | 0 | 0 | 0 | 0 | 0 | d__Eukaryota; k__Fungi; p__Ascomycota; c__Eurotiomycetes; o__Eurotiales; f__Aspergillaceae; g__Penicillium |
| **OTU156** | 0 | 2 | 0 | 0 | 0 | 0 | 0 | 0 | 0 | 0 | d__Eukaryota; k__Fungi; p__Ascomycota; c__Dothideomycetes; o__Capnodiales; f__Mycosphaerellaceae |
| **OTU157** | 4 | 0 | 2 | 3 | 0 | 0 | 0 | 0 | 0 | 0 | d__Eukaryota; k__Fungi |
| **OTU158** | 1 | 1 | 0 | 1 | 0 | 0 | 0 | 0 | 0 | 0 | d__Eukaryota; k__Fungi; p__Ascomycota; c__Leotiomycetes; o__Helotiales |
| **OTU159** | 0 | 1 | 1 | 0 | 0 | 0 | 0 | 0 | 0 | 0 | d__Eukaryota; k__Fungi; p__Ascomycota; c__Saccharomycetes; o__Saccharomycetales; f__Trichomonascaceae; g__Trichomonascus; s__Trichomonascus_ciferrii |
| **OTU160** | 0 | 7 | 0 | 0 | 0 | 0 | 0 | 0 | 0 | 0 | d__Eukaryota; k__Fungi; p__Ascomycota; c__Dothideomycetes; o__Pleosporales; f__Sporormiaceae; g__Preussia; s__Preussia_sp |
| **OTU161** | 0 | 1 | 3 | 0 | 0 | 0 | 0 | 1 | 0 | 0 | d__Eukaryota; k__Fungi; p__Ascomycota; c__Sordariomycetes; o__Hypocreales; f__Nectriaceae |
| **OTU162** | 0 | 0 | 1 | 4 | 0 | 0 | 0 | 0 | 0 | 0 | d__Eukaryota; k__Fungi; p__Ascomycota; c__Saccharomycetes; o__Saccharomycetales; f__Debaryomycetaceae; g__Meyerozyma; s__Meyerozyma_guilliermondii |
| **OTU163** | 0 | 4 | 0 | 0 | 0 | 0 | 0 | 0 | 0 | 0 | d__Eukaryota; k__Fungi; p__Ascomycota; c__Dothideomycetes; o__Capnodiales; f__Davidiellaceae; g__Cladosporium; s__Cladosporium_velox |
| **OTU164** | 0 | 0 | 0 | 1 | 0 | 0 | 0 | 0 | 0 | 0 | d__Eukaryota; k__Fungi; p__Ascomycota |
| **OTU165** | 0 | 30 | 1 | 0 | 0 | 0 | 0 | 0 | 0 | 0 | d__Eukaryota; k__Fungi; p__Ascomycota; c__Sordariomycetes; o__Hypocreales; f__Nectriaceae; g__Fusarium |
| **OTU166** | 0 | 2 | 1 | 4 | 0 | 0 | 0 | 0 | 0 | 0 | d__Eukaryota; k__Fungi; p__Ascomycota; c__Eurotiomycetes; o__unidentified; f__unidentified; g__unidentified; s__Eurotiomycetes_sp |
| **OTU167** | 611 | 53 | 1276 | 1895 | 1 | 0 | 0 | 0 | 0 | 1 | d__Eukaryota; k__Fungi; p__Ascomycota; c__Saccharomycetes; o__Saccharomycetales; f__Incertae_sedis; g__Candida; s__Candida_tropicalis |
| **OTU168** | 0 | 0 | 12 | 3 | 0 | 0 | 0 | 0 | 0 | 0 | d__Eukaryota; k__Fungi; p__Ascomycota |
| **OTU169** | 1 | 1 | 2 | 2 | 0 | 0 | 0 | 0 | 0 | 0 | d__Eukaryota; k__Fungi; p__Basidiomycota; c__Tremellomycetes; o__Tremellales; f__Incertae_sedis |
| **OTU170** | 0 | 0 | 5 | 0 | 0 | 0 | 0 | 0 | 0 | 0 | d__Eukaryota; k__Fungi |
| **OTU171** | 4 | 0 | 7 | 0 | 0 | 0 | 0 | 0 | 0 | 0 | d__Eukaryota; k__Fungi; p__Ascomycota; c__Eurotiomycetes; o__Eurotiales; f__Aspergillaceae; g__Aspergillus; s__Aspergillus_penicillioides |
| **OTU172** | 1 | 3 | 0 | 0 | 0 | 0 | 0 | 0 | 0 | 0 | d__Eukaryota; k__Fungi; p__Ascomycota; c__Eurotiomycetes; o__Eurotiales; f__Aspergillaceae; g__Penicillium; s__Penicillium_olsonii |
| **OTU173** | 0 | 4 | 0 | 0 | 0 | 0 | 0 | 0 | 0 | 0 | d__Eukaryota; k__Fungi; p__Ascomycota |
| **OTU174** | 0 | 0 | 5 | 1 | 0 | 0 | 0 | 0 | 0 | 0 | d__Eukaryota; k__Fungi; p__Basidiomycota; c__Tremellomycetes; o__Tremellales; f__Incertae_sedis; g__Cryptococcus; s__Cryptococcus_curvatus |
| **OTU175** | 0 | 5 | 1 | 0 | 0 | 0 | 0 | 0 | 0 | 0 | d__Eukaryota; k__Fungi; p__Ascomycota; c__Sordariomycetes |
| **OTU176** | 58 | 1 | 18 | 3 | 0 | 0 | 0 | 0 | 0 | 0 | d__Eukaryota; k__Fungi; p__Ascomycota; c__Eurotiomycetes; o__Eurotiales; f__Aspergillaceae; g__Penicillium |
| **OTU177** | 0 | 0 | 3 | 1 | 0 | 0 | 0 | 0 | 0 | 0 | d__Eukaryota; k__Fungi; p__Ascomycota |
| **OTU178** | 0 | 2 | 0 | 0 | 0 | 0 | 0 | 0 | 0 | 0 | d__Eukaryota; k__Fungi; p__Ascomycota; c__Dothideomycetes; o__Pleosporales; f__Sporormiaceae; g__Preussia; s__Preussia_sp |
| **OTU179** | 4 | 4 | 36 | 3 | 0 | 0 | 0 | 0 | 0 | 0 | d__Eukaryota; k__Fungi; p__Ascomycota; c__Eurotiomycetes; o__Eurotiales; f__Aspergillaceae; g__Aspergillus; s__Aspergillus_gracilis |
| **OTU180** | 2 | 0 | 8 | 4 | 0 | 0 | 0 | 0 | 0 | 0 | d__Eukaryota; k__Fungi; p__Ascomycota; c__Incertae_sedis; o__Incertae_sedis; f__Incertae_sedis; g__Hansfordia; s__Hansfordia_sp_XC_2012 |
| **OTU181** | 0 | 0 | 186 | 12 | 0 | 0 | 0 | 0 | 0 | 0 | d__Eukaryota; k__Fungi; p__Ascomycota; c__Saccharomycetes; o__Saccharomycetales; f__Incertae_sedis; g__Candida; s__Candida_orthopsilosis |
| **OTU182** | 0 | 0 | 0 | 4 | 0 | 0 | 0 | 0 | 0 | 0 | d__Eukaryota; k__Fungi; p__Ascomycota; c__Sordariomycetes; o__Hypocreales; f__Incertae_sedis; g__Acremonium; s__Acremonium_sp |
| **OTU183** | 0 | 6 | 0 | 0 | 0 | 0 | 0 | 0 | 0 | 0 | d__Eukaryota; k__Fungi; p__Ascomycota; c__Eurotiomycetes; o__Chaetothyriales; f__Herpotrichiellaceae; g__Phialophora; s__Phialophora_cyclaminis |
| **OTU184** | 24 | 1 | 35 | 107 | 0 | 0 | 0 | 0 | 0 | 0 | d__Eukaryota; k__Fungi; p__Ascomycota; c__Saccharomycetes; o__Saccharomycetales; f__Saccharomycetaceae; g__Kluyveromyces; s__Kluyveromyces_marxianus |
| **OTU185** | 1 | 6 | 0 | 0 | 0 | 0 | 0 | 0 | 0 | 0 | d__Eukaryota; k__Fungi; p__Ascomycota; c__Sordariomycetes; o__Microascales; f__unidentified; g__unidentified; s__Microascales_sp |
| **OTU186** | 0 | 0 | 1007 | 26 | 0 | 1 | 0 | 1 | 0 | 0 | d__Eukaryota; k__Fungi; p__Ascomycota; c__Saccharomycetes; o__Saccharomycetales; f__Incertae_sedis; g__Candida; s__Candida_parapsilosis |
| **OTU187** | 216 | 0 | 0 | 0 | 0 | 0 | 0 | 0 | 0 | 0 | d__Eukaryota; k__Fungi; p__Ascomycota |
| **OTU188** | 0 | 6 | 0 | 0 | 0 | 0 | 0 | 0 | 0 | 0 | d__Eukaryota; k__Fungi; p__Ascomycota; c__Eurotiomycetes; o__Eurotiales; f__Aspergillaceae; g__Penicillium |
| **OTU189** | 1 | 0 | 0 | 0 | 0 | 0 | 0 | 0 | 0 | 0 | d__Eukaryota; k__Fungi; p__Ascomycota; c__Sordariomycetes |
| **OTU190** | 1 | 1 | 3 | 2 | 0 | 0 | 0 | 0 | 0 | 0 | d__Eukaryota; k__Fungi; p__Ascomycota |
| **OTU191** | 0 | 8 | 0 | 0 | 0 | 0 | 0 | 0 | 0 | 0 | d__Eukaryota; k__Fungi; p__Basidiomycota; c__Tremellomycetes; o__Tremellales; f__Incertae_sedis; g__Cryptococcus; s__Cryptococcus_aerius |
| **OTU192** | 0 | 4 | 0 | 0 | 0 | 0 | 0 | 0 | 0 | 0 | d__Eukaryota; k__Fungi; p__Ascomycota |
| **OTU193** | 7 | 0 | 0 | 0 | 0 | 0 | 0 | 0 | 0 | 0 | d__Eukaryota; k__Fungi; p__Ascomycota; c__Eurotiomycetes; o__Eurotiales; f__Aspergillaceae; g__Talaromyces |
| **OTU194** | 0 | 4 | 0 | 0 | 0 | 0 | 0 | 0 | 0 | 0 | d__Eukaryota; k__Fungi; p__Basidiomycota; c__Tremellomycetes; o__Tremellales; f__Incertae_sedis; g__Cryptococcus; s__Cryptococcus_albidus |
| **OTU195** | 0 | 5 | 3 | 0 | 0 | 0 | 0 | 0 | 0 | 0 | d__Eukaryota; k__Fungi; p__Ascomycota; c__Dothideomycetes; o__Capnodiales; f__Mycosphaerellaceae; g__Pseudocercospora |
| **OTU196** | 46 | 3 | 11 | 106 | 0 | 0 | 0 | 0 | 0 | 0 | d__Eukaryota; k__Fungi; p__Basidiomycota; c__Tremellomycetes; o__Trichosporonales; f__Trichosporonaceae; g__Trichosporon; s__Trichosporon_vadense |
| **OTU197** | 1 | 0 | 0 | 1 | 0 | 0 | 0 | 0 | 0 | 0 | d__Eukaryota; k__Fungi; p__Ascomycota |
| **OTU198** | 0 | 0 | 4 | 0 | 0 | 0 | 0 | 0 | 0 | 0 | d__Eukaryota; k__Fungi; p__Basidiomycota; c__Tremellomycetes; o__Tremellales; f__Incertae_sedis; g__Cryptococcus |
| **OTU199** | 0 | 0 | 4 | 2 | 0 | 0 | 0 | 0 | 0 | 0 | d__Eukaryota; k__Fungi; p__Ascomycota; c__Saccharomycetes; o__Saccharomycetales; f__Incertae_sedis; g__Lodderomyces; s__Lodderomyces_elongisporus |
| **OTU200** | 0 | 0 | 10 | 2 | 0 | 0 | 0 | 0 | 0 | 0 | d__Eukaryota; k__Fungi; p__Ascomycota; c__Saccharomycetes; o__Saccharomycetales; f__Trichomonascaceae; g__Blastobotrys; s__Blastobotrys_proliferans |
| **OTU201** | 0 | 0 | 0 | 8 | 0 | 0 | 0 | 0 | 0 | 0 | d__Eukaryota; k__Fungi; p__Ascomycota; c__Sordariomycetes |
| **OTU202** | 0 | 0 | 3 | 0 | 0 | 0 | 0 | 0 | 0 | 0 | d__Eukaryota; k__Fungi; p__Ascomycota |
| **OTU203** | 0 | 6 | 0 | 0 | 0 | 0 | 0 | 0 | 0 | 0 | d__Eukaryota; k__Fungi; p__Ascomycota; c__Leotiomycetes; o__Thelebolales |
| **OTU204** | 0 | 0 | 1 | 0 | 0 | 0 | 0 | 0 | 0 | 0 | d__Eukaryota; k__Fungi; p__Basidiomycota; c__Agaricostilbomycetes; o__Agaricostilbales; f__Agaricostilbaceae; g__Sterigmatomyces; s__Sterigmatomyces_halophilus |
| **OTU205** | 15 | 0 | 0 | 0 | 0 | 0 | 0 | 0 | 0 | 0 | d__Eukaryota; k__Fungi; p__Ascomycota; c__Eurotiomycetes; o__Eurotiales; f__Aspergillaceae; g__Talaromyces |
| **OTU206** | 0 | 0 | 2 | 3 | 0 | 0 | 0 | 0 | 0 | 0 | d__Eukaryota; k__Fungi; p__Ascomycota; c__Eurotiomycetes; o__Eurotiales; f__Thermoascaceae; g__Thermoascus; s__Thermoascus_crustaceus |
| **OTU207** | 0 | 0 | 0 | 2 | 0 | 0 | 0 | 0 | 0 | 0 | d__Eukaryota; k__Fungi; p__Ascomycota |
| **OTU208** | 77 | 57 | 584 | 2333 | 0 | 0 | 0 | 0 | 0 | 1 | d__Eukaryota; k__Fungi; p__Ascomycota; c__Sordariomycetes; o__Hypocreales |
| **OTU209** | 0 | 0 | 2 | 0 | 0 | 0 | 0 | 0 | 0 | 0 | d__Eukaryota; k__Fungi; p__Ascomycota; c__Sordariomycetes; o__Hypocreales; f__Nectriaceae |
| **OTU210** | 0 | 22 | 0 | 0 | 0 | 0 | 0 | 0 | 0 | 0 | d__Eukaryota; k__Fungi; p__Ascomycota; c__Sordariomycetes; o__Hypocreales; f__Bionectriaceae; g__Clonostachys; s__Clonostachys_rosea_f._catenulata |
| **OTU211** | 1 | 0 | 0 | 0 | 0 | 0 | 0 | 0 | 0 | 0 | d__Eukaryota; k__Fungi; p__Ascomycota; c__Dothideomycetes; o__Capnodiales |
| **OTU212** | 1 | 0 | 0 | 5 | 0 | 0 | 0 | 0 | 0 | 0 | d__Eukaryota; k__Fungi; p__Ascomycota; c__Dothideomycetes; o__Pleosporales; f__Lophiostomataceae; g__Lophiostoma; s__Lophiostoma_sp_DMW2181 |
| **OTU213** | 1 | 0 | 5 | 1 | 0 | 0 | 0 | 0 | 0 | 0 | d__Eukaryota; k__Fungi; p__Ascomycota; c__Sordariomycetes; o__Xylariales; f__Amphisphaeriaceae; g__Pestalotiopsis; s__Pestalotiopsis_camelliae |
| **OTU214** | 439 | 33 | 153 | 51 | 0 | 0 | 0 | 0 | 0 | 1 | d__Eukaryota; k__Fungi; p__Ascomycota; c__Eurotiomycetes; o__Eurotiales; f__Aspergillaceae; g__Aspergillus; s__Aspergillus_vitricola |
| **OTU215** | 2 | 0 | 2 | 0 | 0 | 0 | 0 | 0 | 0 | 0 | d__Eukaryota; k__Fungi; p__Ascomycota; c__Dothideomycetes; o__Capnodiales |
| **OTU216** | 4010 | 1817 | 1504 | 595 | 11 | 32 | 15 | 16 | 21 | 29 | d__Eukaryota; k__Fungi; p__Ascomycota; c__Eurotiomycetes; o__Eurotiales; f__Aspergillaceae; g__Aspergillus; s__Aspergillus_cibarius |

**Table-S2 Comparison of microbial diversity estimation and coverage of ITS gene libraries at 97% similarity from the sequencing analysis**

| **Sample** | **No. of reads** | **No. of OTUs** | **Coverage** | **ACE** | **Chao** | **Shannon** | **Simpson** |
| --- | --- | --- | --- | --- | --- | --- | --- |
| **S1** | 35172 | 98 | 0.998704 | 125.9145 | 129.9091 | 2.468022 | 0.130002 |
| **S2** | 27611 | 128 | 0.998800 | 143.5354 | 143.7895 | 1.910574 | 0.258553 |
| **S3** | 23782 | 130 | 0.999136 | 140.3464 | 139.5625 | 2.808219 | 0.088549 |
| **S4** | 37278 | 99 | 0.999040 | 113.6081 | 111.6667 | 2.40271 | 0.135352 |
| **S5** | 32769 | 5 | 0.999856 | 0 | 8 | 0.006087 | 0.998657 |
| **S6** | 24345 | 4 | 0.999904 | 0 | 5 | 0.012533 | 0.996742 |
| **S7** | 20846 | 3 | 0.999952 | 0 | 3 | 0.006453 | 0.998466 |
| **S8** | 33861 | 6 | 0.999856 | 12 | 7.5 | 0.008833 | 0.997986 |
| **S9** | 26465 | 4 | 0.999904 | 0 | 5 | 0.00901 | 0.997795 |
| **S10** | 23055 | 13 | 0.999712 | 38.51922 | 16.75 | 0.021839 | 0.994922 |

**Table-S3 The detail information of Venn diagram analysis (A: group Ⅰ, S1; B: group Ⅱ, S2; C: group Ⅲ, S3-S4; D: group Ⅳ, S5-S10)**

| **Only A** | **A & C** | **A & C & D** | **A & D** | **A & B** | **A & B & C** | **A & B & C & D** | **A & B & D** | **Only B** | **B & C** | **B & C & D** | **B & D** | **Only C** | **C & D** | **Only D** |
| --- | --- | --- | --- | --- | --- | --- | --- | --- | --- | --- | --- | --- | --- | --- |
| OTU18 | OTU6 | OTU74 | **/** | **/** | OTU4 | OTU71 | **/** | OTU2 | OTU25 | **/** | **/** | OTU1 | **/** | OTU105 |
| OTU26 | OTU11 | **/** | **/** | **/** | OTU7 | OTU72 | **/** | OTU9 | OTU29 | **/** | **/** | OTU3 | **/** | **/** |
| OTU35 | OTU19 | **/** | **/** | **/** | OTU12 | OTU108 | **/** | OTU10 | OTU31 | **/** | **/** | OTU5 | **/** | **/** |
| OTU36 | OTU33 | **/** | **/** | **/** | OTU22 | OTU135 | **/** | OTU14 | OTU69 | **/** | **/** | OTU13 | **/** | **/** |
| OTU40 | OTU65 | **/** | **/** | **/** | OTU24 | OTU216 | **/** | OTU16 | OTU83 | **/** | **/** | OTU15 | **/** | **/** |
| OTU55 | OTU93 | **/** | **/** | **/** | OTU30 | **/** | **/** | OTU21 | OTU84 | **/** | **/** | OTU17 | **/** | **/** |
| OTU63 | OTU97 | **/** | **/** | **/** | OTU39 | **/** | **/** | OTU27 | OTU91 | **/** | **/** | OTU20 | **/** | **/** |
| OTU76 | OTU104 | **/** | **/** | **/** | OTU42 | **/** | **/** | OTU38 | OTU98 | **/** | **/** | OTU28 | **/** | **/** |
| OTU77 | OTU136 | **/** | **/** | **/** | OTU45 | **/** | **/** | OTU41 | OTU115 | **/** | **/** | OTU34 | **/** | **/** |
| OTU80 | OTU157 | **/** | **/** | **/** | OTU50 | **/** | **/** | OTU48 | OTU121 | **/** | **/** | OTU44 | **/** | **/** |
| OTU95 | OTU171 | **/** | **/** | **/** | OTU52 | **/** | **/** | OTU54 | OTU122 | **/** | **/** | OTU46 | **/** | **/** |
| OTU111 | OTU176 | **/** | **/** | **/** | OTU53 | **/** | **/** | OTU59 | OTU132 | **/** | **/** | OTU47 | **/** | **/** |
| OTU138 | OTU180 | **/** | **/** | **/** | OTU58 | **/** | **/** | OTU60 | OTU144 | **/** | **/** | OTU51 | **/** | **/** |
| OTU155 | OTU184 | **/** | **/** | **/** | OTU68 | **/** | **/** | OTU61 | OTU149 | **/** | **/** | OTU56 | **/** | **/** |
| OTU187 | OTU215 | **/** | **/** | **/** | OTU75 | **/** | **/** | OTU64 | OTU166 | **/** | **/** | OTU57 | **/** | **/** |
| OTU193 | **/** | **/** | **/** | **/** | OTU81 | **/** | **/** | OTU82 | OTU195 | **/** | **/** | OTU62 | **/** | **/** |
| OTU205 | **/** | **/** | **/** | **/** | OTU86 | **/** | **/** | OTU87 | **/** | **/** | **/** | OTU66 | **/** | **/** |
| / | **/** | **/** | **/** | **/** | OTU88 | **/** | **/** | OTU89 | **/** | **/** | **/** | OTU67 | **/** | **/** |
| / | **/** | **/** | **/** | **/** | OTU100 | **/** | **/** | OTU94 | **/** | **/** | **/** | OTU70 | **/** | **/** |
| / | **/** | **/** | **/** | **/** | OTU109 | **/** | **/** | OTU101 | **/** | **/** | **/** | OTU73 | **/** | **/** |
| / | **/** | **/** | **/** | **/** | OTU114 | **/** | **/** | OTU102 | **/** | **/** | **/** | OTU78 | **/** | **/** |
| / | **/** | **/** | **/** | **/** | OTU128 | **/** | **/** | OTU103 | **/** | **/** | **/** | OTU79 | **/** | **/** |
| / | **/** | **/** | **/** | **/** | OTU139 | **/** | **/** | OTU106 | **/** | **/** | **/** | OTU85 | **/** | **/** |
| / | **/** | **/** | **/** | **/** | OTU140 | **/** | **/** | OTU107 | **/** | **/** | **/** | OTU90 | **/** | **/** |
| / | **/** | **/** | **/** | **/** | OTU143 | **/** | **/** | OTU112 | **/** | **/** | **/** | OTU96 | **/** | **/** |
| / | **/** | **/** | **/** | **/** | OTU145 | **/** | **/** | OTU113 | **/** | **/** | **/** | OTU110 | **/** | **/** |
| / | **/** | **/** | **/** | **/** | OTU150 | **/** | **/** | OTU123 | **/** | **/** | **/** | OTU119 | **/** | **/** |
| / | **/** | **/** | **/** | **/** | OTU154 | **/** | **/** | OTU129 | **/** | **/** | **/** | OTU124 | **/** | **/** |
| / | **/** | **/** | **/** | **/** | OTU167 | **/** | **/** | OTU130 | **/** | **/** | **/** | OTU125 | **/** | **/** |
| / | **/** | **/** | **/** | **/** | OTU179 | **/** | **/** | OTU131 | **/** | **/** | **/** | OTU126 | **/** | **/** |
| / | **/** | **/** | **/** | **/** | OTU196 | **/** | **/** | OTU133 | **/** | **/** | **/** | OTU134 | **/** | **/** |
| / | **/** | **/** | **/** | **/** | OTU208 | **/** | **/** | OTU146 | **/** | **/** | **/** | OTU137 | **/** | **/** |
| / | **/** | **/** | **/** | **/** | OTU214 | **/** | **/** | OTU147 | **/** | **/** | **/** | OTU141 | **/** | **/** |
| / | **/** | **/** | **/** | **/** | **/** | **/** | **/** | OTU156 | **/** | **/** | **/** | OTU151 | **/** | **/** |
| / | **/** | **/** | **/** | **/** | **/** | **/** | **/** | OTU160 | **/** | **/** | **/** | OTU152 | **/** | **/** |
| / | **/** | **/** | **/** | **/** | **/** | **/** | **/** | OTU163 | **/** | **/** | **/** | OTU161 | **/** | **/** |
| / | **/** | **/** | **/** | **/** | **/** | **/** | **/** | OTU165 | **/** | **/** | **/** | OTU162 | **/** | **/** |
| / | **/** | **/** | **/** | **/** | **/** | **/** | **/** | OTU172 | **/** | **/** | **/** | OTU168 | **/** | **/** |
| / | **/** | **/** | **/** | **/** | **/** | **/** | **/** | OTU173 | **/** | **/** | **/** | OTU169 | **/** | **/** |
| / | **/** | **/** | **/** | **/** | **/** | **/** | **/** | OTU175 | **/** | **/** | **/** | OTU170 | **/** | **/** |
| / | **/** | **/** | **/** | **/** | **/** | **/** | **/** | OTU178 | **/** | **/** | **/** | OTU174 | **/** | **/** |
| / | **/** | **/** | **/** | **/** | **/** | **/** | **/** | OTU183 | **/** | **/** | **/** | OTU177 | **/** | **/** |
| / | **/** | **/** | **/** | **/** | **/** | **/** | **/** | OTU185 | **/** | **/** | **/** | OTU181 | **/** | **/** |
| / | **/** | **/** | **/** | **/** | **/** | **/** | **/** | OTU188 | **/** | **/** | **/** | OTU182 | **/** | **/** |
| / | **/** | **/** | **/** | **/** | **/** | **/** | **/** | OTU191 | **/** | **/** | **/** | OTU186 | **/** | **/** |
| / | **/** | **/** | **/** | **/** | **/** | **/** | **/** | OTU192 | **/** | **/** | **/** | OTU190 | **/** | **/** |
| / | **/** | **/** | **/** | **/** | **/** | **/** | **/** | OTU194 | **/** | **/** | **/** | OTU198 | **/** | **/** |
| / | **/** | **/** | **/** | **/** | **/** | **/** | **/** | OTU203 | **/** | **/** | **/** | OTU199 | **/** | **/** |
| / | **/** | **/** | **/** | **/** | **/** | **/** | **/** | OTU210 | **/** | **/** | **/** | OTU200 | **/** | **/** |
| / | **/** | **/** | **/** | **/** | **/** | **/** | **/** | **/** | **/** | **/** | **/** | OTU201 | **/** | **/** |
| / | **/** | **/** | **/** | **/** | **/** | **/** | **/** | **/** | **/** | **/** | **/** | OTU202 | **/** | **/** |
| / | **/** | **/** | **/** | **/** | **/** | **/** | **/** | **/** | **/** | **/** | **/** | OTU206 | **/** | **/** |
| / | **/** | **/** | **/** | **/** | **/** | **/** | **/** | **/** | **/** | **/** | **/** | OTU207 | **/** | **/** |
| / | **/** | **/** | **/** | **/** | **/** | **/** | **/** | **/** | **/** | **/** | **/** | OTU209 | **/** | **/** |
| / | **/** | **/** | **/** | **/** | **/** | **/** | **/** | **/** | **/** | **/** | **/** | OTU212 | **/** | **/** |
| / | **/** | **/** | **/** | **/** | **/** | **/** | **/** | **/** | **/** | **/** | **/** | OTU213 | **/** | **/** |


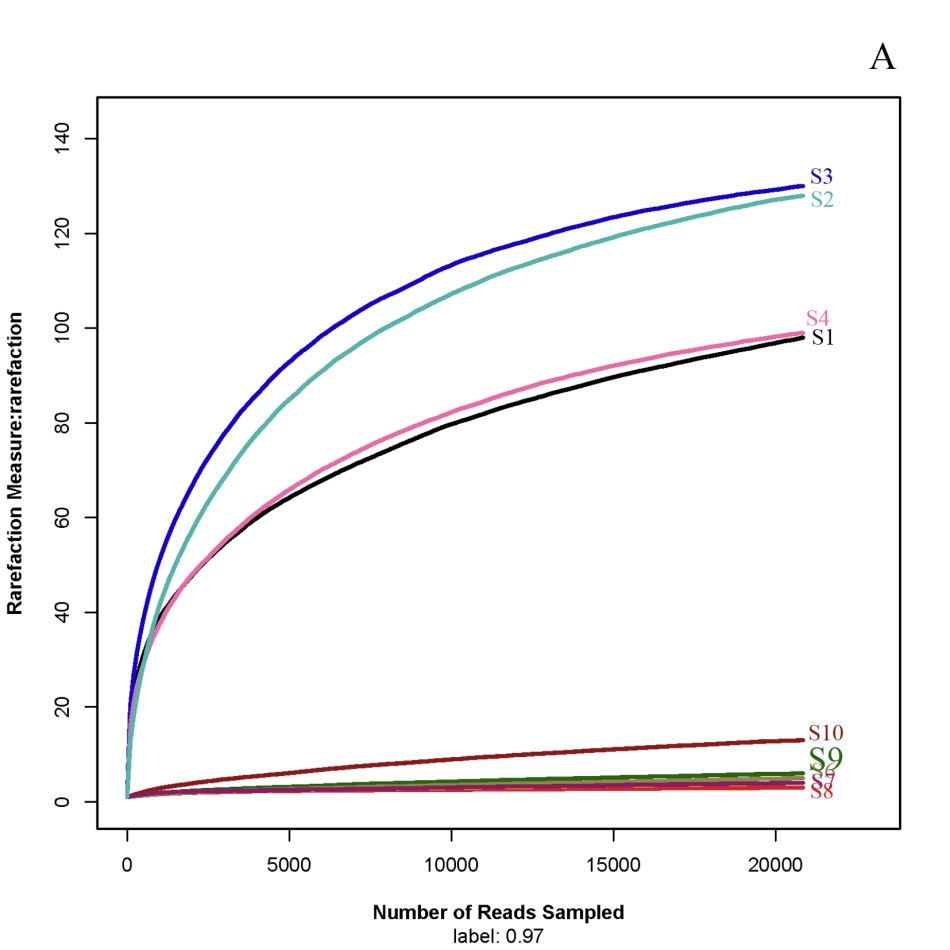

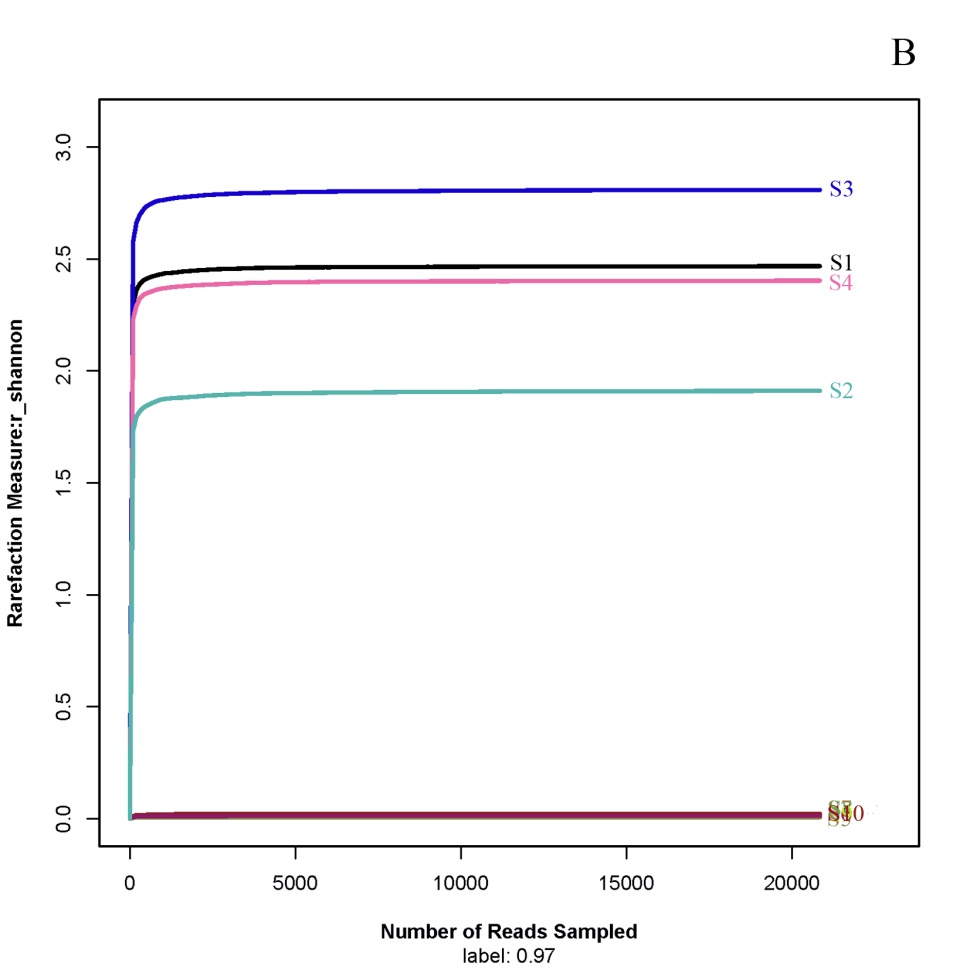


**Figure-S1** The rarefaction and Shannon-Wiener analysis of pyrosequencing tag in microbiota of tea samples from manufacturing process of Fu brick tea. Rarefaction curve (A), Shannon-Wiener curve (B).


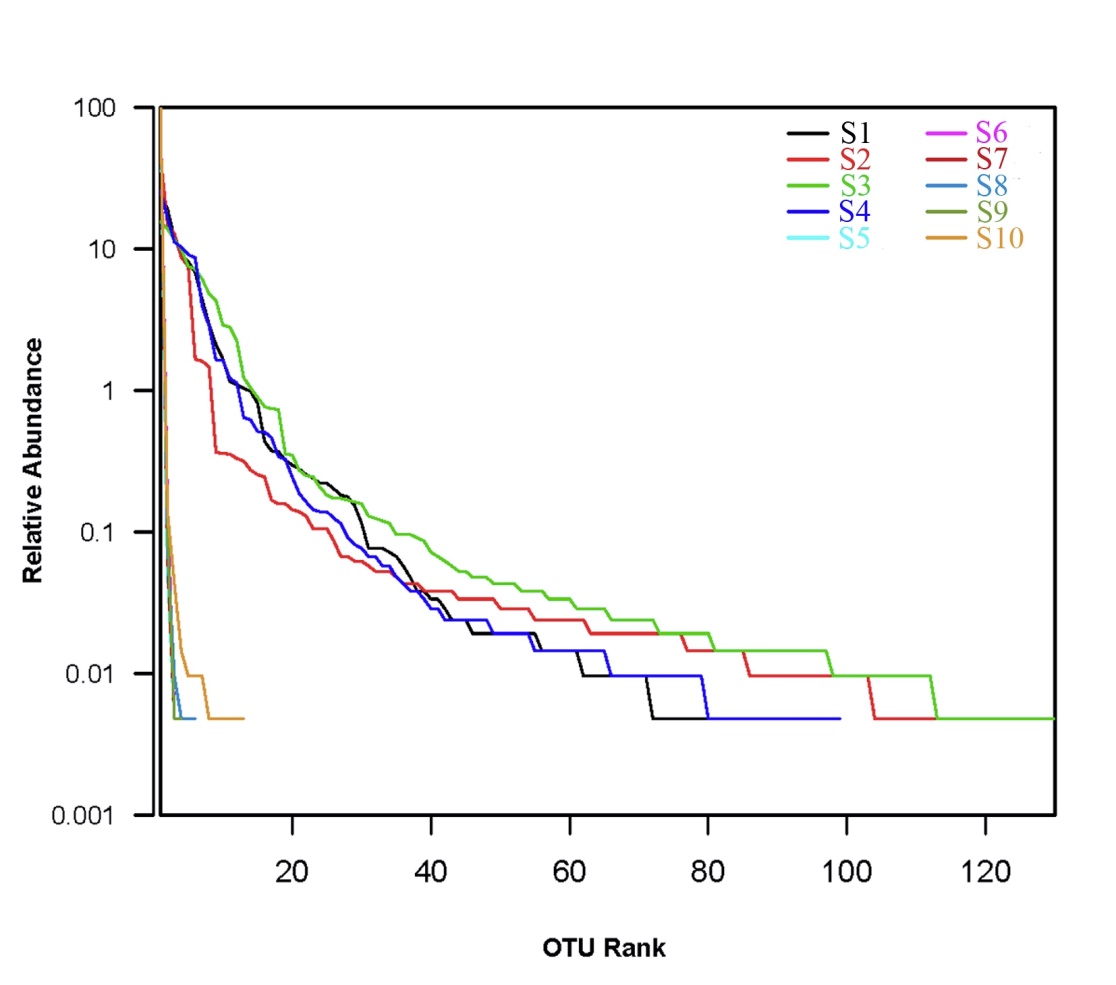


**Figure-S2** Rank abundance curve of fungal OTUs derived from tea samples
